# Predicting Intensive Care Unit admission among patients presenting to the emergency department using machine learning and natural language processing

**DOI:** 10.1371/journal.pone.0229331

**Published:** 2020-03-03

**Authors:** Marta Fernandes, Rúben Mendes, Susana M. Vieira, Francisca Leite, Carlos Palos, Alistair Johnson, Stan Finkelstein, Steven Horng, Leo Anthony Celi

**Affiliations:** 1 IDMEC, Instituto Superior Técnico, Universidade de Lisboa, Lisbon, Portugal; 2 Hospital da Luz Learning Health, Lisbon, Portugal; 3 Hospital Beatriz Ângelo, Luz Saúde, Lisbon, Portugal; 4 MIT Critical Data, Laboratory for Computational Physiology, Harvard-MIT Health Sciences & Technology, Massachusetts Institute of Technology, Cambridge, Massachusetts, United States of America; 5 Institute for Data, Systems and Society, Massachusetts Institute of Technology, Cambridge, Massachusetts, United States of America; 6 Department of Emergency Medicine / Division of Clinical Informatics / Center for Healthcare Delivery Science, Beth Israel Deaconess Medical Center, Boston, Massachusetts, United States of America; 7 Division of Pulmonary Critical Care and Sleep Medicine, Beth Israel Deaconess Medical Center, Boston, Massachusetts, United States of America; Liverpool John Moores University, UNITED KINGDOM

## Abstract

The risk stratification of patients in the emergency department begins at triage. It is vital to stratify patients early based on their severity, since undertriage can lead to increased morbidity, mortality and costs. Our aim was to present a new approach to assist healthcare professionals at triage in the stratification of patients and in identifying those with higher risk of ICU admission. Adult patients assigned Manchester Triage System (MTS) or Emergency Severity Index (ESI) 1 to 3 from a Portuguese and a United States Emergency Departments were analyzed. Variables routinely collected at triage were used and natural language processing was applied to the patient chief complaint. Stratified random sampling was applied to split the data in train (70%) and test (30%) sets and 10-fold cross validation was performed for model training. Logistic regression, random forests, and a random undersampling boosting algorithm were used. We compared the performance obtained with the reference model—using only triage priorities—with the models using additional variables. For both hospitals, a logistic regression model achieved higher overall performance, yielding areas under the receiver operating characteristic and precision-recall curves of 0.91 (95% CI 0.90-0.92) and 0.30 (95% CI 0.27-0.33) for the United States hospital and of 0.85 (95% CI 0.83-0.86) and 0.06 (95% CI 0.05-0.07) for the Portuguese hospital. Heart rate, pulse oximetry, respiratory rate and systolic blood pressure were the most important predictors of ICU admission. Compared to the reference models, the models using clinical variables and the chief complaint presented higher recall for patients assigned MTS/ESI 3 and can identify patients assigned MTS/ESI 3 who are at risk for ICU admission.

## Introduction

Emergency departments (EDs) often form the front line of health care systems and play a critical role in ensuring an efficient and quality service for patients with acute conditions [1]. The first evaluation, where the patient condition and acuity level are defined, is performed at triage, which has emerged as a method to identify patients who need immediate care.

According to a report published in 2017 by the Agency for Healthcare Research and Quality (AHRQ) [2], there were 120 million ED visits in 2006 in the United States and by 2014, there were 137.8 million ED visits, an increase of 14.8 percent. In 2015, the Organisation for Economic Co-operation and Development (OECD) Health Committee publish a report stating that the number of ED visits across OECD countries was about 31 per 100 population in 2011. The number of visits per capita was the highest in Portugal, with over 70 visits per 100 population [1]. Thus, the case study of Portugal is relevant, given that it represents high demand for emergency services, compared to other OECD countries.

The Manchester Triage System (MTS) and the Emergency Severity Index (ESI) are 5-level triage systems widely used in Europe and in the US, respectively. In ESI, treatment priority is decided on the basis of disease severity and the expected resource needs [3]. This system uses an algorithm, with ratings ranging from level 1 (patients with life-threatening conditions) to level 5 (the least resource-intensive patients). MTS priorities range from level 1 (emergent patients that should have immediate medical observation) to level 5 (non urgent patients that should wait a maximum time of 4 hours for medical observation).

Recent studies have shown good results in prediction of hospital admission [4–12], ED LOS [13], ICU admission [9, 14], mortality [9, 15, 16] and combined outcome of mortality and ICU admission [9, 14, 17] using machine learning techniques and historical information accessible from the EHR of triaged patients. There are also contributions of prediction models for triage classification in the literature [18–22]. Among the predictors used in the referred studies were age, gender, arrival mode, vital signs acquired at triage, chief complaint, time of admission, patient comorbidities and relevant medical history.

In this work, we employed machine learning to identify ED patients with high risk of ICU admission. We used data routinely collected at triage from the EDs of Hospital Beatriz Ângelo (HBA) in Portugal and of Beth Israel Deaconess Medical Center (BIDMC) in the United States. The primary outcome was admission to the ICU or equivalent in the first 24 hours after triage, accounting for differences in clinical practice across the two sites. At BIDMC, the outcome measure was admission to ICU. At HBA, the outcome measure consisted of admission to ICU or Intermediate Care Unit where medical or surgical patients who need ongoing monitoring are admitted. BIDMC does not have an intermediate care unit; all equivalent patients are admitted to the ICU. The models were developed for the cohort of patients assigned MTS/ESI 1 to 3. The patients assigned MTS/ESI 4 to 5 present to the ED for minor issues such as rashes or minor lacerations, and rarely are admitted to the ICU. We excluded these less urgent patients to reduce the class imbalance. We then compared the performance against a reference model trained only with the triage priority assigned to patients, with ESI or MTS, from the United States and Portuguese hospitals, respectively.

## Materials and methods

### Data acquisition

Data were acquired from the Emergency Department Information Systems (EDIS) of a Portuguese and a United States hospital. The data ranges from 2012 to 2016 and from 2011 to 2016, for the Portuguese and United States data, respectively, with a total of 599276 and 267257 ED visits in the adult population (≥ 18 years old). This study was approved by HBA Ethics Committee. The use of the BIDMC data was approved by the Institutional Review Board (IRB) under protocol number 2011P-000356. The HBA Ethics Committee and the BIDMC IRB waived the requirement for informed consent.

### Predictors

For modelling, we included the variables routinely collected at triage (vital signs—temperature, heart rate, respiratory rate, systolic blood pressure, diastolic blood pressure, mean arterial blood pressure, pulse oximetry SpO_2_ and pain scale), the chief complaint, glycemia levels, Glasgow coma scale (GCS), the triage priority assigned to the patient, the patient age and gender, mode of arrival to the ED (ambulance, walk-in), disabilities (stretcher, wheelchair or none), time of triage (weekday, hour and month), ED visit (first triage registered on the system or not), prescription of complementary means of diagnostic at triage (number of exams), and type of exams prescribed (ophthalmology, otolaryngology, electrocardiogram, X-ray and orthopedic).

### Outcomes

The outcomes considered as outputs for the models were the following, in a period within 24 hours after triage:

- **BIDMC**: ICU—admission to ICU
- **HBA**: ICU&INT—admission to ICU or to Intermediate Care Unit

### Inclusion and exclusion criteria

At HBA, re-triage is performed when a patient is triaged again after the initial triage for new assessment of parameters, change of priority, activation of clinical pathways, or introduction/correction of other information in the registry. Re-triages were excluded in the dataset for modelling, given that not all patients are re-triaged. There were patients which were transferred from HBA ED to another hospital ED. There was no information of outcome for these patients, therefore patients referred to another ED within the 24 hours were excluded. Patients who died before ED admission were excluded as well. Finally, patients assigned MTS/ESI 4 to 5 were excluded, as well as patients assigned a white priority (in HBA this priority is assigned for patients with less urgency for care), since the study focused on patients assigned MTS/ESI 1 to 3. For detailed exclusion criteria refer to S1 Appendix in supplementary materials.

### Modeling

#### Modeling design

We used stratified random sampling to split the dataset into train (70%) and test (30%) sets so that class labels were balanced in each dataset. The dataset was pre-processed before the modeling stage, which can be depicted in supplementary materials (S2 Appendix). We performed a stratified 10 fold cross validation (CV) in the training set to perform a randomized search for hyperparameter optimization. The information regarding hyperparameter tuning is depicted in S1 Table. The configuration of the model with highest AUROC was selected as well as the corresponding threshold. This model was evaluated in the held-out test dataset. We performed 100 iterations of bootstrapping random sampling in 95% confidence intervals (CI) to measure variance in performance. The methodology for modeling can be depicted in S1 Fig.

#### Modeling techniques

We used logistic regression (LR) with L2 regularization, RUSBoost and random forests regression bootstrap aggregation of decision trees (RFR). These algorithms were selected so we could compare a boosting classification technique—RUSBoost, and a bagging regression technique—random forests with a more traditional technique—LR.

LR is a general statistical model originally developed by Joseph Berkson [23]. The prediction $\hat{y}$ or probability of an event for certain input features values *x*, is related to the *N* input features according to (1), with parameters *β*_0_, *β*_1_, … *β*_*N*_.

$$\begin{array}{c} \hat{y}\left(x\right)=\frac{1}{1+e^{-(\beta_{0}+\beta_{1}x+...+\beta_{N}x)}} \end{array}$$ (1)

RUSBoost is a well known boosting algorithm that uses a combination of RUS (random under-sampling) and the standard Adaptive Boosting (AdaBoost) procedure, and can improve the learning when using imbalanced data [24]. AdaBoost is adaptive in the sense that subsequent weak learners are adjusted in favor of those instances misclassified by previous classifiers. RUSBoost randomly removes examples from the majority class until the desired balance is achieved. This technique presents the advantage of being extremely fast to train the models, compared to other techniques such as LR and random forests, since the training dataset size is reduced. Denoting $\hat{y}$ as the boosting classifier prediction, with *T* as the total number of classifiers, each *e*_*t*_ is a weak learner that takes an object *x* as input and returns a value indicating its class. A set of weights ϖ is assigned for the T classifiers, in order to take a weighted average of their estimates. A learner with a good classification result will be assigned a higher weight than a poor one.

$$\begin{array}{c} \hat{y}\left(x\right)=\sum_{t=1}^{T}\varpi_{t}e_{t}\left(x\right) \end{array}$$ (2)

Random forests [25] perform a randomized sampling process to train a set of individual decision trees, aggregating the output to produce a single probabilistic prediction for each outcome. For classification tasks, the random forests classifier outputs the class which is voted more times by the individual trees. For regression tasks it gives the mean prediction of the individual trees as indicated in (3).

$$\begin{array}{c} \hat{y}\left(x\right)=\frac{1}{T}\sum_{t=1}^{T}e_{t}\left(x\right) \end{array}$$ (3)

We also applied a multimodeling approach with a majority voting classifier, where the voting was performed with all the predicted labels from the base learners, and the final prediction was made using the label with most votes. The decision criterion to select which models should vote for the final classification consisted on their individual performance, namely their sensitivity. Multimodeling approaches have been used in the literature [26–31] and a more comprehensive analysis of multimodeling and ensemble techniques can be found in [32].

#### Natural language processing

The chief complaint for BIDMC dataset is essentially semi-structured text mapped to SNOMED-CT using the HierArchical Presenting Problem ontologY (HaPPy) [33], which improves the quality of this feature. For this feature, contractions were fixed, punctuation was removed, words were set to lowercase and tokenized. We also performed abbreviation expansion, replaced numbers by words, removed stopwords and finally applied lemmatization. The chief complaint for HBA dataset consists of unstructured free written text and it was subjected to lowercasing, a process of temporal normalization, tokenization, abbreviations expansion and correction using Jaro-Winkler and stemming. More detailed preprocessing can be depicted in S2 Appendix.

To include the chief complaint as model predictor, the Term frequency–inverse document frequency (TF-idf) was used for text vectorization. Tf-idf is a numerical statistic that reflects how important a word is to a document in a collection or corpus and it was first introduced in [34]. The Tf–idf value increases proportionally to the number of times an N-gram (in the present case—a word) appears in a document and is offset by the number of documents in the corpus that contain the word. This process automatically adjusts the weighting of words that appear more frequently and which might have less meaning. The term frequency (tf) in Tf-idf expressed in (4) indicates how frequently a word appears in the document, measuring the local importance of it. The term inverse document frequency (idf) of each word is expressed in (5) and it measures the rareness of a term. Tf-idf is the product of tf and idf as expressed in (6).

$$\begin{array}{c} tf\left(N-gram\right)=\frac{Number\;of\;times\;the\;N-gram\;appears\;in\;the\;Document}{Number\;of\;N-grams\;in\;the\;Document} \end{array}$$ (4)

$$\begin{array}{c} idf\left(N-gram\right)=\log_{10}(\frac{Number\;of\;documents}{Number\;of\;documents\;containing\;the\;N-gram}) \end{array}$$ (5)

$$\begin{array}{c} Tf-idf(N-gram)=tf(N-gram)\times idf(N-gram) \end{array}$$ (6)

The number of N-grams (words) to select from each patient chief complaint as well as the total number of words to use from the training vocabulary are indicated in S1 Table.

#### Performance measures

According to [35], one of the most commonly reported measures for validating modeling performance, is the area under the receiver-operating characteristic curve (AUROC). This is a function of the true positive ratio or recall versus the false positive ratio (FPR), integrated over all thresholds. FPR in (8) corresponds to a false alarm ratio of the model and represents the cases where the patient is incorrectly classified as positive. Recall in (7) corresponds to the sensitivity of the model and represents the cases where the patient is correctly classified as being positive. An AUROC of 0.50 is achieved through random predictions where 1 represents a perfect discrimination. The pair (Recall_*k*_, FPR_*k*_) is referred to as an operating point for this curve.

The area under the precision-recall curve (AUPRC) was also assessed and it is a useful measure of success of prediction when the classes are very imbalanced. The AUPRC shows the trade-off between precision in (9) and recall in (7) for different thresholds. A high area under the curve represents both high recall and high precision. The pair (Recall_*k*_, Precision_*k*_) is referred to as an operating point for this curve. Other measures for assessing the modeling performance were the specificity or true negative rate (TNR) in (10), precision or positive predictive value (PPV) in (9) and accuracy in (11), which were used in previous studies [4–6]. We also assessed F1-score in (12), a measure that displays the trade-off between recall and precision, suited for dealing with imbalanced datasets [13]. We used Cohen’s Kappa (*κ*) [36] to analyze inter-rater reliability, which represents the agreement between two variables [37]. Cohen’s Kappa is presented in (13) where *p*_*o*_ is the empirical probability of agreement on the label assigned to a sample, and *p*_*e*_ is the expected agreement when both raters assign labels randomly. Finally, we present the standardized mortality ratio (SMR) which in this case represents the ratio between the observed number of positive outcomes (ICU admission) predicted by the model and the number of positive outcomes which would be expected.

$$\begin{array}{c} Recall=\frac{TP}{TP+FN}; \end{array}$$ (7)

$$\begin{array}{c} FPR=\frac{FP}{FP+TN}; \end{array}$$ (8)

$$\begin{array}{c} Precision=\frac{TP}{TP+FP}; \end{array}$$ (9)

$$\begin{array}{c} Specificity=\frac{TN}{TN+FP}; \end{array}$$ (10)

$$\begin{array}{c} Accuracy=\frac{TN+TP}{TN+TP+FN+FP}; \end{array}$$ (11)

$$\begin{array}{c} F-score=\frac{2\times TP}{2\times TP+FN+FP}. \end{array}$$ (12)

$$\begin{array}{c} \kappa =\frac{p_{0}-p_{e}}{1-p_{e}}. \end{array}$$ (13)

Where TN and TP indicate the true negatives and positives, patients that were correctly identified as belonging to class 0 and 1, respectively; FN and FP indicate the false negatives and positives, patients that were incorrectly identified as belonging to class 0 and 1, respectively.

## Results

### Emergency department data

**Fig 1:**
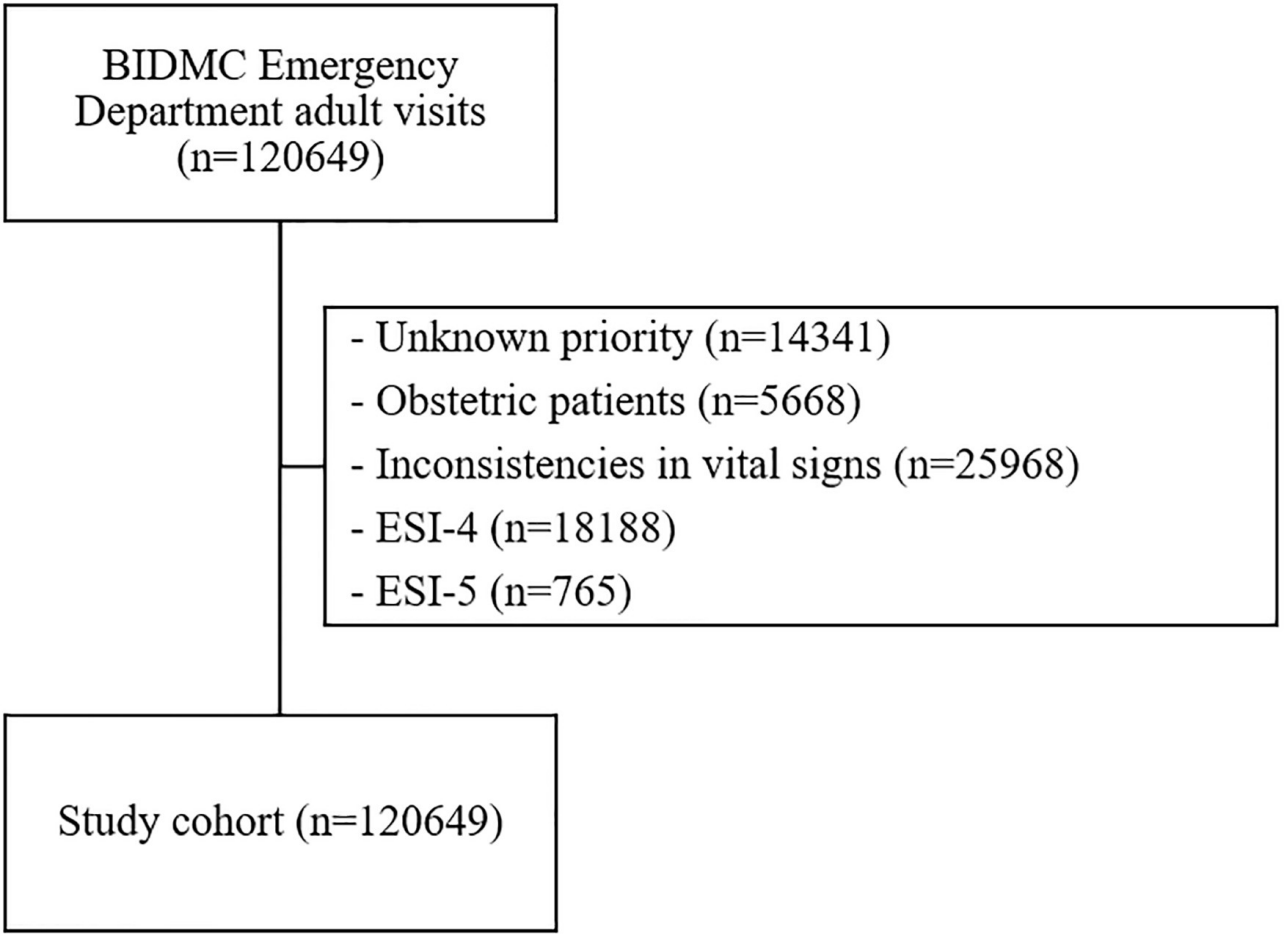
Inclusion and exclusion criteria for Beth Israel Deaconess Medical Center dataset. “n” corresponds to the number of triages.

In the BIDMC data with a population of 267257 adult ED visits, we excluded triages with unknown priority (n = 14341), obstetric patients (n = 5668), inconsistencies in vital signs (n = 25968), ESI-4 (n = 18188) and ESI-5 (n = 765) leaving a cohort of 120649 triaged patients, as presented in Fig 1. This cohort was comprised of 7.0% ESI-1, 34.2% ESI-2 and 58.8% ESI-3 patients. Among patients admitted to the ICU in the first 24 hours after triage (3426–2.8%), there were 35.9% ESI-1, 51.5% ESI-2 and 12.5% ESI-3.

**Fig 2:**
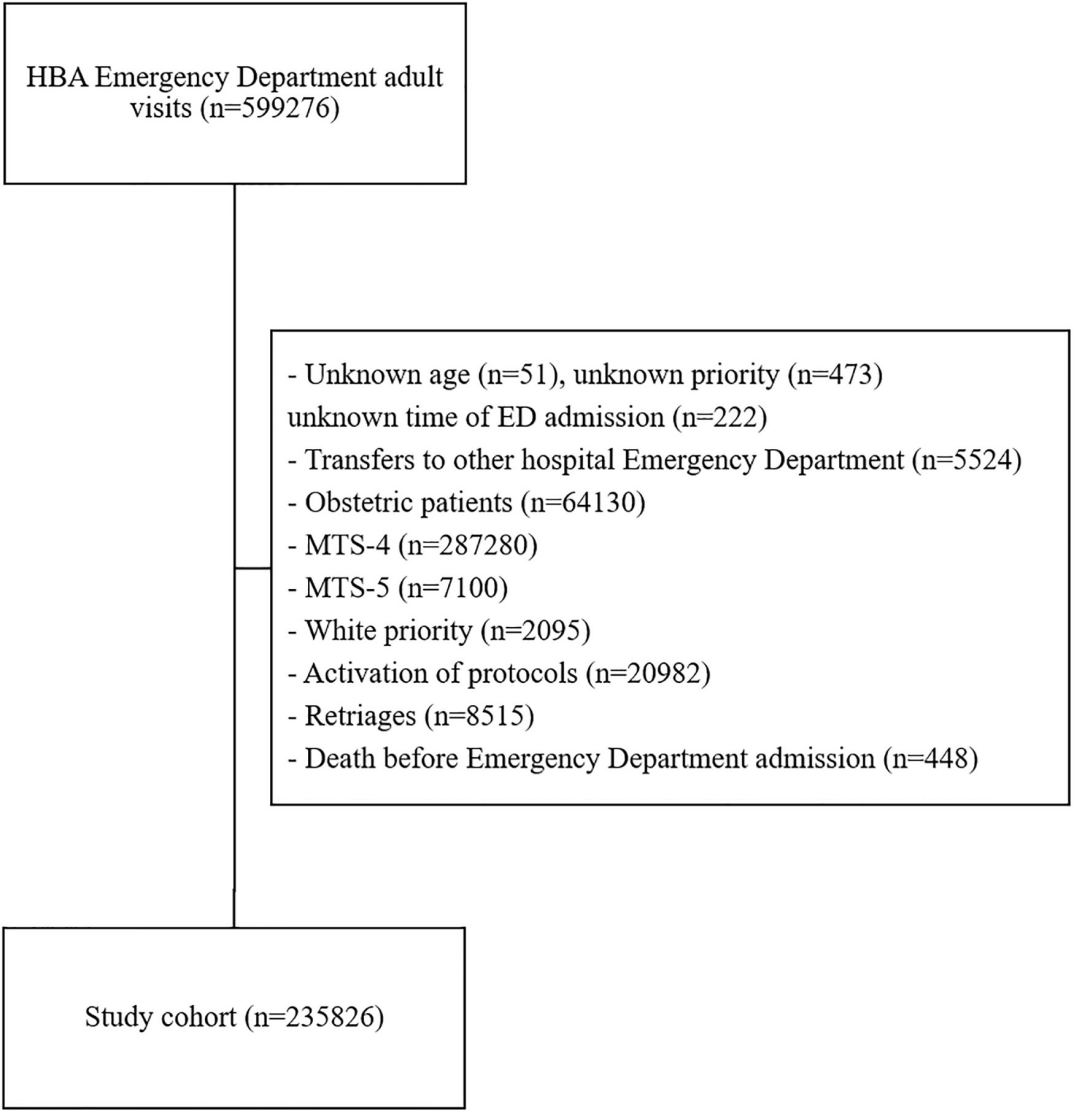
Inclusion and exclusion criteria for Hospital Beatriz Ângelo dataset. “n” corresponds to the number of triages.

In the HBA data with a population of 599276 adult ED visits, we excluded triaged patients with unknown age (n = 51), unknown priority (n = 473), unknown time of ED admission (n = 222), transfers to other hospital ED (n = 5524), obstetrics (n = 64130), MTS-4 (n = 287280), MTS-5 (n = 7100), white priority (n = 2095), activation of protocols (n = 20982), re-triages (n = 8515) and death before ED admission (n = 448) leaving a cohort of 235826 triaged patients, as presented in Fig 2. This cohort was comprised of 0.6% MTS-1, 17.5% MTS-2 and 81.9% MTS-3 patients. Among patients admitted to the ICU and Intermediate Care Unit in the first 24 hours after triage (1784–0.8%), there were 9.8% MTS-1, 53.3% MTS-2 and 37.0% MTS-3.

Demographics and a subset of variables are presented in Table 1. A list with all the variables used for modeling is presented in S2 Table. The descriptive statistics of all predictors used for modeling are presented in S3, S4 and S5 Tables. For both hospitals, the gender was balanced and triaged population had a median age of 59 and 51 years old in HBA and BIDMC datasets. In HBA dataset, the top five most common triage discriminators were moderate pain (35.0%), correspondent to a level of pain between 5 and 7; pleuritic pain (6.0%); sudden onset (5.2%) e.g. evidence of stroke; low pulse oximetry (4.7%) where 90% ≤ SpO_2_ ≤ 95%; and severe pain (4.6%) correspondent to a level of pain between 8 and 10. In BIDMC dataset, according to ICD-9 and ICD-10 codes assignment at triage, the top five most common conditions were chest pain (4.2%), abdominal pain (2.6%), syncope (2.0%), headache (2.0%) and pneumonia (1.4%). For both hospitals, the admitted patients were older with an average of 65 years old and there were more male patients being admitted than female. Compared to non-admitted, admitted patients to BIDMC and HBA ICU presented an average of: 2 and 1 breaths per minute higher; 9 and 5 beats per minute higher; 1% and 2% lower pulse oximetry; 7 and 3 mmHg lower mean arterial blood pressure. The average temperature of 37 degrees Celsius was the same for both admitted and not admitted patients in both hospitals.

**Table 1:** Demographic variables and a subset of features available for both hospitals are summarized for each cohort of emergency department patients.

Variable (units)	BIDMC		HBA	
	ICU admission	No ICU admission	ICU admission	No ICU admission
Age (years old)	65 (19-93)	50 (19-93)	65 (18-101)	58 (18-108)
Female gender	1594 (47)	62502 (53)	757 (42)	130145 (56)
Male gender	1832 (53)	54721 (47)	1027 (58)	103903 (44)
Vital signs				
Respiratory rate (breaths/min)	19 (6-40)	17 (0-40)	18 (6-40)	17 (0-40)
Heart rate (beats/min)	93 (14-190)	84 (12-234)	91 (24-220)	86 (0-293)
Temperature (°C)	37 (20-41)	37 (20-42)	37 (27-41)	37 (20-42)
Pulse oximetry (%)	97 (50-100)	98 (58-100)	94 (55-100)	96 (50-100)
Systolic blood pressure (mmHg)	126 (47-263)	135 (24-270)	138 (53-260)	143 (36-292)
Diastolic blood pressure (mmHg)	72 (16-214)	77 (6-191)	75 (25-140)	77 (6-201)
Mean Arterial Blood Pressure (mmHg)	90 (35-222)	97 (34-204)	96 (37-173)	99 (27-211)
Triage priorities				
Emergent (ESI-1, MTS-1)	1231 (36)	7173 (6)	174 (10)	1189 (1)
Very urgent (ESI-2, MTS-2)	1766 (52)	39540 (34)	950 (53)	40266 (17)
Urgent (ESI-3, MTS-3)	429 (12)	70510 (60)	660 (37)	192593 (82)
Outcome	3426 (2.8)	117223 (97.2)	1784 (0.8)	234048 (99.2)

The table shows number of patients. The figures in parentheses are the column percentages within each categorical variable for the respective outcome of admission. For continuous variables mean and range are presented. BIDMC—Beth Israel Deaconess Medical Center. HBA—Hospital Beatriz Ângelo. ESI—Emergency Severity Index. MTS—Manchester Triage System. ICU—Intensive Care Unit.

### Prediction of ICU admission in BIDMC

**Fig 3:**
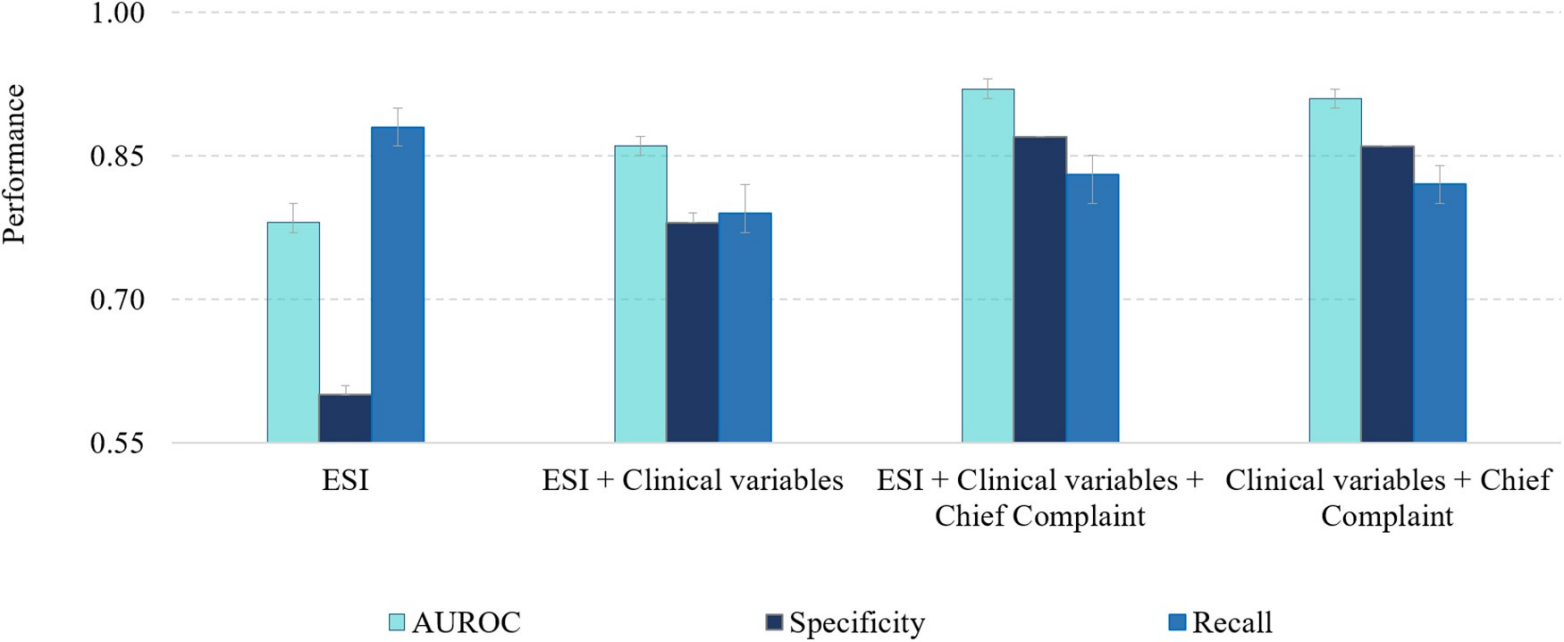
Performance of regularized logistic regression in test using the different subsets of predictors for Beth Israel Deaconess Medical Center dataset. ESI—Emergency Severity Index, AUROC—area under the ROC curve.

For prediction of ICU admission among BIDMC patients assigned the ESI 1 to 3, we had a training set with control/exposure groups of 82056/2398 patients, and the test set with 35167/1028 patients. The modeling results for ESI and additional features can be depicted in S7 Table and visualized graphically in Fig 3. Since the assignment of triage priority is subjective and can be variable across institutions, the model selected for further analysis was developed with all available predictors except for the triage priority. The additional features added to the BIDMC model were information on the number of abnormal and missing vital signs, mean arterial blood pressure, information regarding time of triage (weekday, hour and month) and the chief complaint.

Regarding the measure of F1-score and the AP, the values were relatively low due to the class imbalance present in the data (S7 Table). Analyzing the performance results obtained when adding clinical variables to ESI, the performance was overall higher when compared to the reference model using only ESI. Compared to RUSBoost or RFR, the LR model consistently presented higher sensitivity. RFR models had the tendency to overfit, while RUSBoost model presented higher F1-score results but at the cost of much lower sensitivity. When adding the chief complaint to the model using clinical variables, there was a significant increase in overall performance.

**Fig 4:**
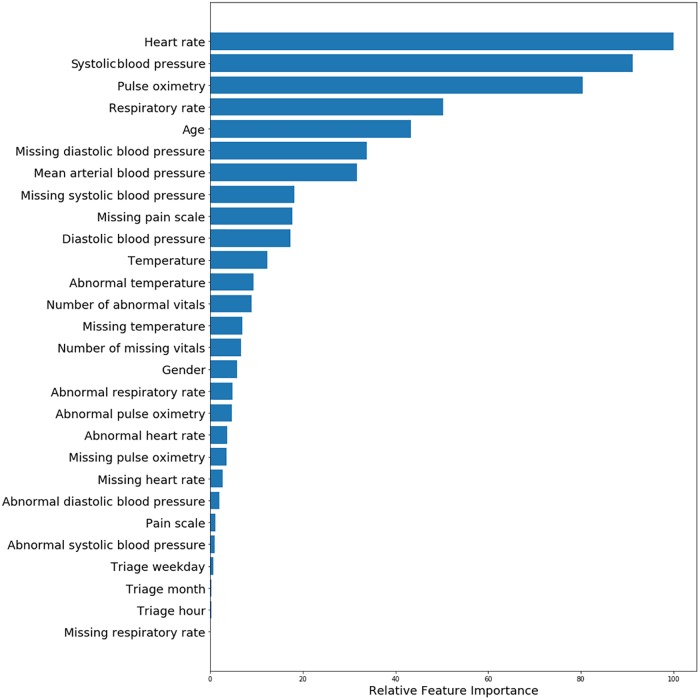
Relative importance of predictors of Intensive Care Unit admission for Beth Israel Deaconess Medical Center dataset obtained with regularized logistic regression using all available variables except triage priority.

We assessed importance estimates of predictors through the absolute values of the coefficients from LR, as presented in Fig 4. The most important predictor was heart rate, followed by systolic blood pressure with a contribution in importance estimates of 90%, pulse oximetry with 80%, respiratory rate with 50% and the patient’s age with 45%. The mean arterial blood pressure contributed an importance estimate of approximately 30% and the remaining predictors less than 20%. The time of triage, weekday and month contributed the least importance estimate of less than 1%.

### Prediction of ICU and Intermediate Care Unit admission in HBA

**Fig 5:**
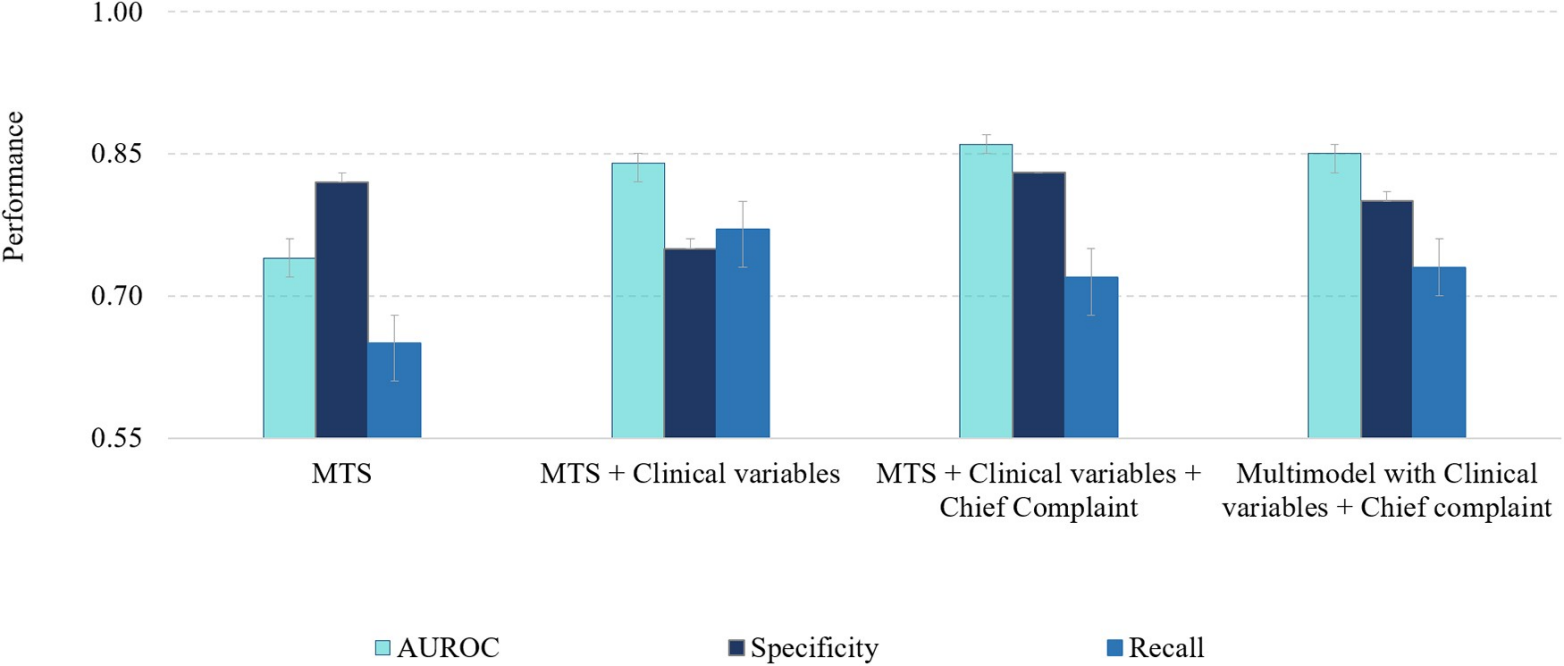
Performance of regularized logistic regression in test using the different subsets of predictors for Hospital Beatriz Ângelo dataset. MTS—Manchester Triage System, AUROC—area under the ROC curve.

For prediction of ICU and Intermediate Care Unit admission among HBA patients assigned the MTS 1 to 3, we had a training set with control/exposure groups of 165082/1249 patients, and in the test set, 70750/535 patients. The modeling results for MTS and additional features can be depicted in S7 Table and visualized graphically in Fig 5. The F1-score and the precision values were low due to class imbalance. We observed that when adding the chief complaint, the sensitivity of the model decreased and the specificity increased. Therefore, a multi-model was created based on a voting classifier between the model using the chief complaint as predictor and the model not using this predictor. The multi-model could achieve a more balanced sensitivity and specificity. Since the assignment of triage priority is subjective, the multi-model consisted of the combination of models using all predictors except for the triage priority.

**Fig 6:**
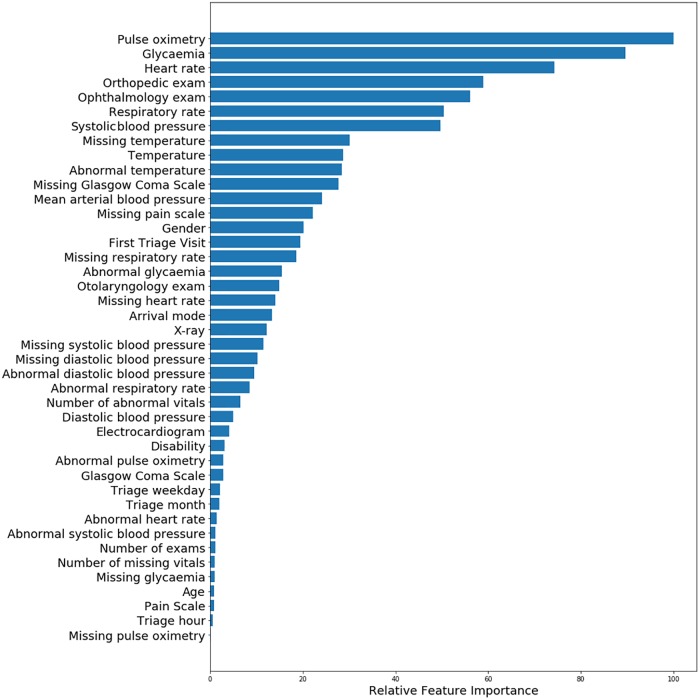
Relative importance of predictors of Intensive Care Unit and Intermediate Care Unit admission for Hospital Beatriz Ângelo dataset, obtained with regularized logistic regression using all available variables except triage priority. Exams are prescribed at the time of triage.

We assessed importance estimates of predictors through the absolute values of the coefficients given by LR, as presented in Fig 6. The most important predictor was the pulse oximetry, followed by glycaemia with a contribution in importance estimates of 90%, heart rate with 75%, orthopedic and ophthalmology consultations with 60% and 55%, respiratory rate with 50% and systolic blood pressure with 50%. The remaining predictors presented an importance estimate less than 30%. The time of triage, weekday and month contributed an importance estimate of less than 5%. These importance estimates are consistent with those of the BIDMC model, with the exception of age. The patient’s age was ranked with a low importance of approximately 1% in the HBA model.

### Models assessment and calibration

The models performance and calibration for both hospitals were assessed. Calibration curves, also referred to as reliability diagrams, present the fraction of patients in the positive class against the predicted probabilities. The mean of the predicted probabilities was computed for each decile. A well calibrated binary classifier will have an increasing number of true cases as one goes from the decile with the lowest mean predicted probability to the decile with the highest mean predicted probability. The performance and information regarding model parameters can be depicted in Table 2 where a comparison between the calibrated and non calibrated models is shown. The calibration curves for both models can be depicted in S2 Fig. Furthermore, we assessed the calibration curves for the different subsets of predictors for each hospital in S3 Fig.

**Table 2:** Modeling results comparison between calibrated models.

	BIDMC model with no priority		HBA multi-model with no priority	
	No calibration	Isotonic calibration	No calibration	Isotonic calibration
AUROC	0.91 [0.90-0.92]	0.91 [0.90-0.92]	0.85 [0.83-0.86]	0.85 [0.83-0.86]
AUPRC	0.32 [0.28-0.35]	0.30 [0.27-0.33]	0.06 [0.05-0.07]	0.06 [0.05-0.07]
AP	0.31 [0.28-0.35]	0.30 [0.27-0.33]	0.06 [0.05-0.07]	0.06 [0.05-0.07]
Specificity	0.86 [0.86-0.87]	0.86 [0.86-0.86]	0.81 [0.80-0.81]	0.85 [0.84-0.85]
Recall	0.81 [0.79-0.84]	**0.82 [0.80-0.84]**	**0.73 [0.7-0.76]**	0.68 [0.65-0.72]
SMR	5.44 [5.1-5.78]	5.62 [5.28-5.97]	26.27 [24.27-28.5]	25.66 [23.68-27.84]
Threshold	0.035	0.030	0.009	0.007
N-gram range	Combination of unigrams and bigrams		M1: unigrams	
Words from vocabulary	9500		M1: 29000	
Warm start	No		Yes	
Regularization constant	1		1	

M1 corresponds to model which uses chief complaint for prediction. In brackets is the result for 100 bootstrapping iterations in 95% confidence intervals. The models selected have recall highlighted in bold. BIDMC—Beth Israel Deaconess Medical Center. HBA—Hospital Beatriz Ângelo. AUROC—area under the ROC curve. AUPRC—area under the precision recall curve. AP—Average precision. SMR—Standardized Mortality Ratio.

For both hospitals, we observed an improvement in calibration as more features were included to the model. For HBA dataset, even in the best calibrated models, the risk of ICU admission was still over-estimated. This is acceptable for this specific use case, as it is better to over-estimate rather than under-estimate probabilities of ICU admission for the high-risk group of patients.

**Fig 7:**
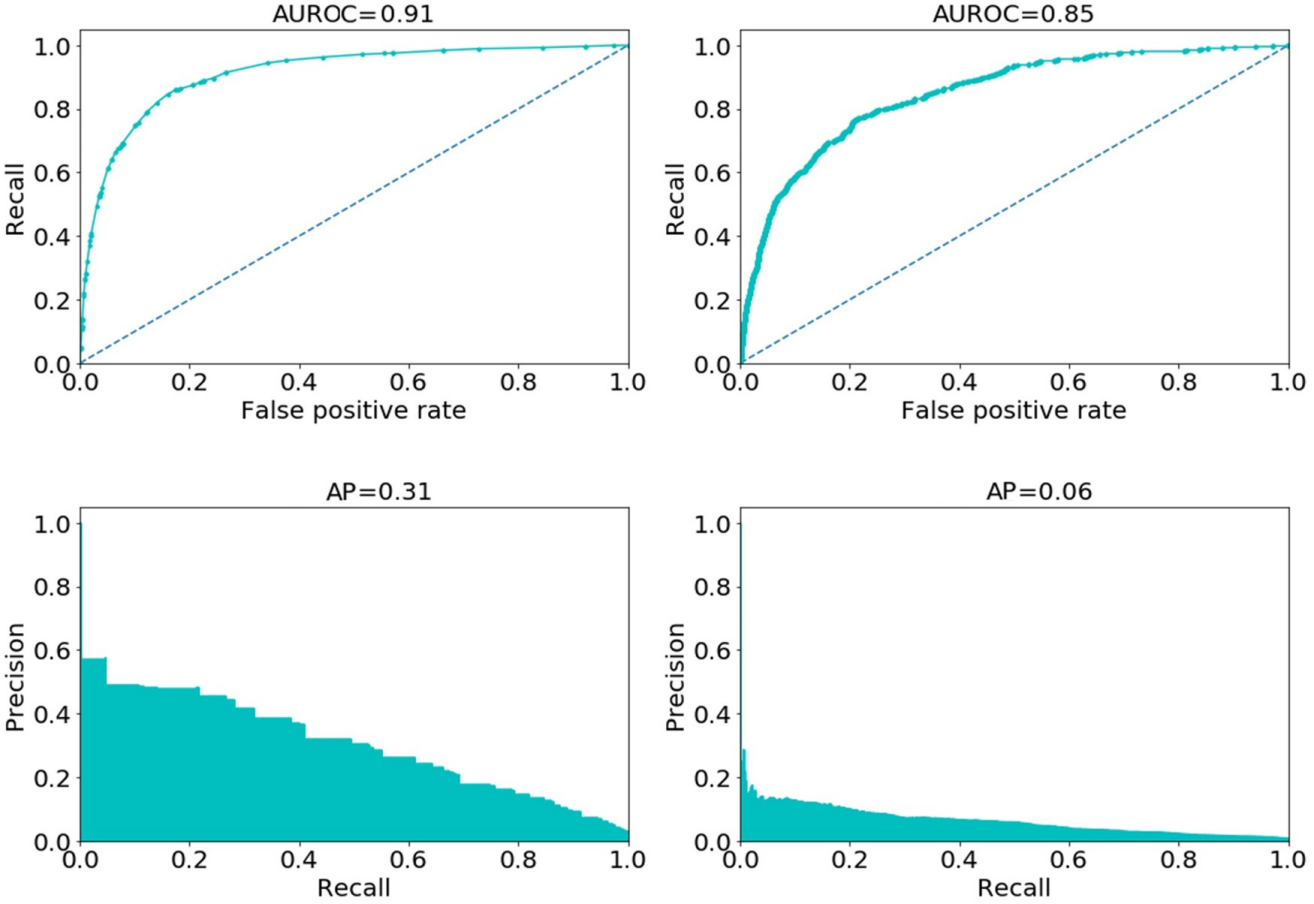
Performance of the model with isotonic calibration for Beth Israel Deaconess Medical Center (on the left) and of the multi-model with no calibration for Hospital Beatriz Ângelo (on the right) using in both logistic regression with all predictors except priority. AUROC—Area under the ROC curve. AUPRC—Area under the precision recall curve.

The AUROC and AUPRC for the BIDMC model with isotonic calibration and the HBA multi-model with no calibration are presented in Fig 7. For HBA, the multi-model without calibration was selected since it presented higher recall than the one with calibration.

Prediction models using clinical variables and the chief complaint were developed for both hospitals. For BIDMC, a model developed with clinical variables and the chief complaint presented lower recall in the identification of patients admitted to the ICU assigned ESI 1 and 2. However, the model was able to identify patients assigned an ESI-3 who were admitted to the ICU, contrary to the reference ESI (S4 Fig). For HBA, we developed a multi-model combining the model developed with clinical variables and the chief complaint with a model developed only with clinical variables so a balance between sensitivity and specificity could be attained. The scenario for HBA was similar as the one of BIDMC. For MTS 1 and 2 the multi-model presented lower recall in the identification of patients admitted to the ICU, however it had the ability to identify patients assigned an MTS-3 who were admitted to the ICU, contrary to the reference MTS (S5 Fig).

## Discussion

In this work, we developed models to predict risk of admission to the ICU at time of emergency department triage in two hospitals, one in Portugal and another in the US. Regularized logistic regression, random forests regression and random undersampling boosting of decision trees were used. The important predictors among patients assigned MTS/ESI 1 to 3 were identified. The performance of the models was compared to the reference model using only the ESI (for BIDMC) and MTS (for HBA) priority. The discrimination and calibration of the models were presented.

Since the assignment of triage priority is subjective and can be variable across institutions, the final models were developed using all clinical variables and the chief complaint, with the exception of triage priority. For both hospitals, heart rate, pulse oximetry, respiratory rate and systolic blood pressure ranked highly for prediction of ICU admission by the LR models. The model using semi-structured data from BIDMC for the chief complaint selected about a third of the words in the training vocabulary compared to the model using unstructured data from HBA. Although the free-text chief complaint may encode additional information, the amount of information compared to the structured chief complaint data could not be measured. For future work we propose to perform feature selection using the chief complaint and analyze the importance of this feature.

For BIDMC, a model developed with clinical variables and the chief complaint with isotonic calibration presented higher overall performance. For HBA, a multi-model combining the model developed with clinical variables and the chief complaint with a model developed only with clinical variables achieved a good balance between recall and specificity. The model for BIDMC data exhibited good calibration properties while for the HBA data, the multi-model over-estimated this risk. We concluded that for this case, an over-estimation of the probabilities of ICU admission for the high-risk group of patients can be better than an under-estimation of the risk. The models presented a higher recall in the identification of patients admitted to the ICU in MTS/ESI-3 priority level while the reference MTS/ESI presented higher recall for MTS/ESI 1 and 2 priority levels. The low measures of F1-score and precision were due to the class imbalance present in the data.

In a similar study [9] several machine learning models were developed to predict a critical outcome of admission to ICU or in-hospital death and their performance was compared with that of the ESI. The machine learning models outperformed the ESI reference model (e.g., AUROC, 0.86 (95%CI 0.85–0.87) in a deep neural network vs 0.74 (95%CI 0.72–0.75) in the reference model). The most important predictors of the critical outcome were patient’s age, respiratory rate, heart rate, systolic blood pressure, pulse oximetry and arrival by ambulance. When compared to the BIDMC model, the same variables were identified as the most important predictors. In another study [14], a LR model was developed to predict a critical outcome of admission to ICU or in-hospital death in a cohort of patients aged 75 and older. The most important predictors were respiratory rate, systolic blood pressure, pulse oximetry and the Glasgow Coma Score. Except for the Glasgow Coma Score—which was not available in the BIDMC dataset, the same vital signs were ranked as the most important predictors.

In a third paper [8], a triage tool called “e-triage” was developed using random forests to predict the need for critical care, an emergency procedure, and inpatient hospitalization. The e-triage models had an AUROC ranging from 0.73 to 0.92 and demonstrated equivalent or improved prediction of patient outcomes compared with ESI at different EDs. Similar to this study, the models developed for BIDMC and HBA were able to predict ICU admission at the time of ED triage among patients assigned MTS/ESI-3 priority level. These results demonstrate an opportunity to complement the already existing triage systems with machine learning models and avoid under-triaging.

## Supporting information

S1 Appendix Exclusion criteria detailed. (PDF)

S2 Appendix Data pre-processing. (PDF)

S1 Table Hyperparameter optimization in random search cross validation. (PDF)

S2 Table Variables used for modelling Hospital Beatriz Ângelo (HBA) and Beth Israel Deaconess Medical Center (BIDMC) emergency department data. (*) Additional predictors available only for HBA dataset.
(PDF)

S3 Table Demographics and vital signs variables used for modelling Hospital Beatriz Ângelo and Beth Israel Deaconess Medical Center emergency department data. The table shows number of patients. The figures in parentheses are the column percentages within each categorical variable for the respective outcome of admission. For continuous variables mean and range are presented.
(PDF)

S4 Table Statistics of the additional Hospital Beatriz Ângelo predictor variables used for modelling. The table shows number of patients. The figures in parentheses are the column percentages within each categorical variable for the respective outcome of admission.
(PDF)

S5 Table Additional variables used for modelling both hospitals emergency departments data. The table shows number of patients. The figures in parentheses are the column percentages within each categorical variable for the respective outcome of admission.
(PDF)

S6 Table Criteria for outlier exclusion and abnormal values identification. DBP—diastolic blood pressure. (1) values not within normal range.
(PDF)

S7 Table Average modeling performance results in test. In brackets is the result for 100 bootstrapping iterations in 95% confidence intervals.
(PDF)

S1 Fig Methodology steps for modeling. AUROC—area under the ROC curve, AUPRC—area under the precision recall curve, TNR—true negative rate or specificity. RUSBoost—Random undersampling boosting algorithm. Tf-idf—Term frequency–inverse document frequency.
(TIF)

S2 Fig Calibration curves of the logistic regression model with isotonic calibration for Beth Israel Deaconess Medical Center (on top) and of the multi-model for Hospital Beatriz Ângelo (on bottom), using all available predictors. Annotations with labels are presented only for the selected models.
(TIF)

S3 Fig Calibration curves for Beth Israel Deaconess Medical Center (on the right) and for Hospital Beatriz Ângelo (on the left) according to the different subsets of modeling predictors. (TIF)

S4 Fig Confusion matrix for the models using data from Beth Israel Deaconess Medical Center. (TIF)

S5 Fig Confusion matrix for the models using data from Hospital Beatriz Ângelo. (TIF)

S6 Fig (TIF)
