# Supplementary material for: Predicting Intensive Care Unit admission among patients presenting to the emergency department using machine learning and natural language processing
Source: PLoS One. 2020 Mar 3;15(3):e0229331. doi: 10.1371/journal.pone.0229331 (PMC7053743; doi:10.1371/journal.pone.0229331)
Supplement: S1 Appendix — (PDF) [file pone.0229331.s001.pdf]

Inclusion and exclusion criteria detailed

For HBA dataset, there were duplicated triages, where triage information of patients were registered more than once in the same episode, with exactly the same parameters values. In these cases, a new registry (and TriageID) was assigned to the patient due to, for e.g., a change of triage priority, activation of a specific clinical pathway, correction of number of days of the chief complaint or the chief complaint itself. For our study, these duplicated triages would introduce a bias in the modelling results, since the model would be trained and tested with data from different triageIDs but with the same parameters information for the same patient. Therefore, the criterion for deletion of duplicated triages consisted on excluding same parameters in a 24 hour period for the same patient, in the respective episode. Patients with activation of clinical protocols (sepsis, stroke, trauma, thoracic pain, anti-hypertensive, analgesia, antipyretic and critical patient protocol specific from HBA) at the triage were excluded from the study, since this was a special cohort of patients with specific guidelines for intervention. Obstetric patients were excluded as well. Patients with data inconsistencies such as unknown age, unknown priority, unknown time of admission in the ED or vital signs such as systolic blood pressure, diastolic blood pressure, oximetry and respiratory rate above 300 mmHg, 200 mmHg, 100% and 80 breaths/minute, respectively, were excluded from both datasets.

Obstetric patients were excluded from BIDMC dataset based on the International Classification of Diseases, Ninth Revision (ICD-9) and Tenth Revision (ICD-10) codes.
