# Supplementary material for: Predicting Intensive Care Unit admission among patients presenting to the emergency department using machine learning and natural language processing
Source: PLoS One. 2020 Mar 3;15(3):e0229331. doi: 10.1371/journal.pone.0229331 (PMC7053743; doi:10.1371/journal.pone.0229331)
Supplement: S2 Appendix — (PDF) [file pone.0229331.s002.pdf]

Data preprocessing

In this paper, the input data consisted of clinical and demographic variables collected at triage in the emergency department. Data pre-processing consisted in outliers processing, missing data imputation and data normalization using NumPy and Pandas in Python version 3.6. To reduce data redundancy and improve data integrity, min-max scaler normalization was used for continuous input variables. The chief complaint for BIDMC dataset was pre-processed as follows: contractions were fixed (e.g. "the patient isn't", replaced by "the patient is not") and punctuation was removed. Words were set to lowercase and tokenized. Abbreviation expansion consisted of replacing abbreviations for their respective medical terms (e.g. "fx" was replaced by fracture and "hx" by history). Numbers were replaced by words, since numbers might be typed in a numeric or alphabetic manner. Stopwords were removed using the dictionary of stopwords from package *nltk*, except for the following terms, which could be relevant in this case: again, before, below, down, no, not, once, only. Duplicated tokens in each patient chief complaint were removed, to further reduce the modeling computational time. Lemmatization was applied to the BIDMC chief complaint. The chief complaint for HBA was subjected to lowercasing, a process of temporal normalization, tokenization, abbreviations expansion and correction using Jaro-Winkler and stemming. The RLSP stemmer was used since it makes less both understemming and overstemming errors when compared to the Portuguese version of Porter's algorithm. A corpus was created for the HBA dataset recurring to the Firefox dictionary for language tokens as well as sources for sublanguage [1] and for medications [2]. For the BIDMC dataset, the corpus was created based on the chief complaint vocabulary after applying the already mentioned pre-processing steps. The chief complaints in HBA are essentially unstructured free written text, while for BIDMC they are semi-structured mapped to SNOMED-CT using the chief complaint ontology HaPPy [5].

The criteria for outliers and abnormal values assessment for each physiological parameter was based on values not within the parameter's physiological range (S6). Dummy indicator variables were created for each input variable, where 1 was imputed each time an abnormal value was identified in the correspondent variable for the patient. Values outside the physiological ranges were identified, but not excluded. A heart rate with a null value corresponded to a cardiopulmonary arrest, therefore it is a possible value and it was not excluded. Only extreme outliers values out of the physiological ranges, that would not be possible to exist, were removed from the dataset. The criteria for outlier exclusion and abnormal values identification was based on established medical physiological ranges [3, 4, 6].

Since for the majority of patients the clinical variables are not all measured at the point of triage, we have missing values in our dataset. Several of these missing values can be classified as Missing Not At Random (MNAR). This can be either because the triage decision makers consider that there is no need to measure all the variables, e.g. patients with lower degree of severity, or because the intervention has to be immediate, and there is no time for assessment of all variables. The criterion for missing data imputation consisted on filling the missing value with real information available collected in a time frame [-60, 15] min, with triage assessment as reference (0 min). The values could have been registered in a different time of the triage assessment (e.g. system down - values registered on paper and later on the information system), so the last present value was imputed. For the remaining missing values, the mean value of the triaged population for each variable was imputed.

References

1. Association of Medical Doctors of Portugal, https://www.medicosdeportugal.pt/glossario/.

2. Infarmed National Authority of Medications and Health Products,http://www.infarmed.pt/.

3. Plus Medline;. https://medlineplus.gov/vitalsigns.html.

4. Medscape; https://emedicine.medscape.com/article/2172054-overview.

5. Horng, Steven and Greenbaum, Nathaniel R and Nathanson, Larry A and McClay, James C and Goss, Foster R and Nielson, Jeffrey A. Consensus Development of a Modern Ontology of Emergency Department Presenting Problems – The Hierarchical Presenting Problem Ontology (HaPPy). Applied clinical informatics. 2019; 10(03):409–420.

6. Salmasi V, Maheshwari K, Yang D, Mascha EJ, Singh A, Sessler DI, et al. Relationship between Intraoperative Hypotension, Defined by Either Reduction from Baseline or Absolute Thresholds, and Acute Kidney and Myocardial Injury after Noncardiac Surgery A Retrospective Cohort Analysis. Anesthesiology: The Journal of the American Society of Anesthesiologists. 2017;126(1):47–65.

7. Avriel M. Nonlinear programming: analysis and methods. Courier Corporation; 2003.
