# Supplementary material for: Predicting Intensive Care Unit admission among patients presenting to the emergency department using machine learning and natural language processing
Source: PLoS One. 2020 Mar 3;15(3):e0229331. doi: 10.1371/journal.pone.0229331 (PMC7053743; doi:10.1371/journal.pone.0229331)
Supplement: S1 Table — (PDF) [file pone.0229331.s003.pdf]

Table S1. Hyperparameter optimization in random search cross validation.

Technique	Hyperparameters
LR	Maximum number of iterations was set to 1000.<br>The limited-memory solver using Broyden Fletcher Goldfarb Shanno algorithm [7] and L2 penalization.<br>The LR regularization constant was varied for the values 0.0001, 0.001, 0.01, 0.1, 1.0.
RUSBoostClassifier	Number of trees was varied for the values 50, 100 and the learning rate for the values 0.1, 1.0.
Random forests	Number of trees was varied for the values 50, 100.<br>The maximum tree depth was varied between 1, 2 and all nodes being expanded until all leaves were pure or contained less than 2 samples to split.<br>The creation of trees was varied between using bootstrap samples or the whole data to fit the model.
TF-IDF	The number of words in a patient's chief complaint was varied between unigrams (a single word), bigrams (two consecutive words) and trigrams (three consecutive words), as following: unigrams, bigrams, triagrams, unigrams + bigrams, bigrams + trigrams and unigrams + bigrams + trigrams.<br>The maximum number of words to consider from the vocabulary was varied for the values {1000, 9500, 15000, 25000, 29000} as well as considering all the vocabulary in train.
