# Supplementary material for: Predicting Intensive Care Unit admission among patients presenting to the emergency department using machine learning and natural language processing
Source: PLoS One. 2020 Mar 3;15(3):e0229331. doi: 10.1371/journal.pone.0229331 (PMC7053743; doi:10.1371/journal.pone.0229331)
Supplement: S2 Table — (*) Additional predictors available only for HBA dataset. (PDF) [file pone.0229331.s004.pdf]

Table S2. Variables used for modelling HBA and BIDMC emergency department data.

**Variable (units)**	
Age (years old)	Pain Scale (0 - 10)
Gender	Pain scale missing (yes/no)
Respiratory rate (breaths/min)	Heart rate (beats/min)
Respiratory rate missing (yes/no)	Heart rate missing (yes/no)
Abnormal respiratory rate (yes/no)	Abnormal heart rate (yes/no)
Temperature (°C)	Pulse oximetry (%)
Temperature missing (yes/no)	Pulse oximetry missing (yes/no)
Abnormal temperature (yes/no)	Abnormal pulse oximetry (yes/no)
Systolic blood pressure (mmHg)	Diastolic blood pressure (mmHg)
Systolic blood pressure missing (yes/no)	Diastolic blood pressure missing (yes/no)
Abnormal systolic blood pressure (yes/no)	Abnormal diastolic blood pressure (yes/no)
Arrival mode (walk-in/ambulance/other) (*)	Glasgow Coma Scale (3 - 15) (*)
Number of exams (0 - 3 or more) (*)	First triage visit (yes/no) (*)
Ophthalmology exam (yes/no) (*)	Triage hour (1-12am)
Otolaryngology exam (yes/no) (*)	Triage weekday (Monday-Sunday)
Electrocardiogram exam (yes/no) (*)	Triage month (January-December)
X-ray exam (yes/no) (*)	Glycemia (mg/dL) (*)
Orthopedic exam (yes/no) (*)	Glycemia missing (yes/no) (*)
Disability (none/stretcher/wheelchair) (*)	Abnormal glycemia (yes/no) (*)
Number of abnormal vital signs (0 - 5)	Mean Arterial Blood Pressure (mmHg)
Triage chief complaint (text)	

(*) Additional predictors available only for HBA dataset.
