# Supplementary material for: Predicting Intensive Care Unit admission among patients presenting to the emergency department using machine learning and natural language processing
Source: PLoS One. 2020 Mar 3;15(3):e0229331. doi: 10.1371/journal.pone.0229331 (PMC7053743; doi:10.1371/journal.pone.0229331)
Supplement: S3 Table — The table shows number of patients. The figures in parentheses are the column percentages within each categorical variable for the respective outcome of admission. For continuous variables mean and range are presented. (PDF) [file pone.0229331.s005.pdf]

Table S3. Demographics and vital signs variables used for modelling HBA and BIDMC emergency department data.

Variable (units)	BIDMC ICU		HBA ICU	
	Admission	No admission	Admission	No admission
Age (years old)	65 (19-93)	50 (19-93)	65 (18-101)	58 (18-108)
Female gender	1594 (47)	62502 (53)	757 (42)	130145 (56)
Male gender	1832 (53)	54721 (47)	1027 (58)	103903 (44)
Respiratory rate (breaths/min)	19 (6-40)	17 (0-40)	18 (6-40)	17 (0-40)
Heart rate (beats/min)	93 (14-190)	84 (12-234)	91 (24-220)	86 (0-293)
Temperature (°C)	37 (20-41)	37 (20-42)	37 (27-41)	37 (20-42)
Pulse oximetry (%)	97 (50-100)	98 (58-100)	94 (55-100)	96 (50-100)
Systolic blood pressure (mmHg)	126 (47-263)	135 (24-270)	138 (53-260)	143 (36-292)
Diastolic blood pressure (mmHg)	72 (16-214)	77 (6-191)	75 (25-140)	77 (6-201)
Mean Arterial Blood Pressure (mmHg)	90 (35-222)	97 (34-204)	96 (37-173)	99 (27-211)
Respiratory rate missing				
1 (yes)	526 (15)	3493 (3)	1077 (60)	191383 (82)
0 (no)	2900 (85)	113730 (97)	707 (40)	42665 (18)
Heart rate missing				
1 (yes)	402 (12)	2241 (2)	502 (28)	114563 (49)
0 (no)	3024 (88)	114982 (98)	1282 (72)	119485 (51)
Temperature missing				
1 (yes)	653 (19)	4597 (4)	444 (25)	50498 (22)
0 (no)	2773 (81)	112626 (96)	1340 (75)	183550 (78)
Pulse oximetry missing				
1 (yes)	490 (14)	3350 (3)	706 (40)	145556 (62)
0 (no)	2936 (86)	113873 (97)	1078 (60)	88492 (38)
Systolic blood pressure missing				
1 (yes)	418 (12)	2770 (2)	922 (52)	167206 (71)
0 (no)	3008 (88)	114453 (98)	862 (48)	66842 (29)
Diastolic blood pressure missing				
1 (yes)	425 (12)	2942 (3)	924 (52)	167508 (72)
0 (no)	3001 (88)	114281 (97)	860 (48)	66540 (28)
Abnormal respiratory rate				
1 (yes)	783 (23)	11260 (10)	1365 (77)	24934 (11)
0 (no)	2643 (77)	105963 (90)	419 (23)	209114 (89)
Abnormal heart rate				
1 (yes)	1299 (38)	56087 (48)	888 (50)	65913 (28)
0 (no)	2127 (62)	61136 (52)	896 (50)	168135 (72)
Abnormal temperature				
1 (yes)	127 (4)	1325 (1)	106 (6)	6801 (3)
0 (no)	3299 (96)	115898 (99)	1678 (94)	227247 (97)
Abnormal pulse oximetry				
1 (yes)	467 (14)	4150 (4)	584 (33)	22526 (10)
0 (no)	2959 (86)	113073 (96)	1200 (67)	211522 (90)
Abnormal systolic blood pressure				
1 (yes)	1041 (30)	41274 (35)	384 (22)	31830 (14)
0 (no)	2385 (70)	75949 (65)	1400 (78)	202218 (86)
Abnormal diastolic blood pressure				
1 (yes)	1195 (35)	29749 (25)	339 (19)	19843 (8)
0 (no)	2231 (65)	87474 (75)	1445 (81)	214205 (92)

The table shows number of patients. The figures in parentheses are the column percentages within each categorical variable for the respective outcome of admission. For continuous variables mean and range are presented.
