# Supplementary material for: Predicting Intensive Care Unit admission among patients presenting to the emergency department using machine learning and natural language processing
Source: PLoS One. 2020 Mar 3;15(3):e0229331. doi: 10.1371/journal.pone.0229331 (PMC7053743; doi:10.1371/journal.pone.0229331)
Supplement: S4 Table — The table shows number of patients. The figures in parentheses are the column percentages within each categorical variable for the respective outcome of admission. (PDF) [file pone.0229331.s006.pdf]

Table S4. Additional variables used for modelling HBA emergency department data.

Variable (units)	HBA ICU Admission	No admission
Arrival mode		
1 (Walk-in)	455 (25)	124535 (53.2)
2 (Ambulance)	866 (49)	56868 (24.3)
3 (Other)	463 (26)	52645 (22.5)
Glasgow Coma Scale		
3	13 (0.7)	110 (0.05)
4	6 (0.3)	23 (0.01)
5	3 (0.2)	37 (0.02)
6	14 (0.8)	135 (0.06)
7	17 (1.0)	218 (0.09)
8	12 (0.7)	402 (0.17)
9	21 (1.2)	784 (0.33)
10	32 (1.8)	1324 (0.57)
11	16 (0.9)	1545 (0.66)
12	19 (1.1)	1307 (0.56)
13	28 (1.6)	1435 (0.61)
14	112 (6.3)	7335 (3.13)
15	1491 (83.6)	219393 (93.74)
Number of exams		
0	1720 (96.4)	212462 (90.8)
1	57 (3.2)	14736 (6.3)
2	4 (0.2)	4650 (2.0)
3 or more	3 (0.2)	2200 (0.9)
Ophthalmology exam		
1 (yes)	1784 (100)	2247 (1)
0 (no)	0 (0)	231801 (99)
Otolaryngology exam		
1 (yes)	1781 (99.8)	487 (0.2)
0 (no)	3 (0.2)	233561 (99.8)
Electrocardiogram exam		
1 (yes)	51 (3)	4016 (2)
0 (no)	1733 (97)	230032 (98)
X-ray exam		
1 (yes)	1784 (100)	104 (0.04)
0 (no)	0 (0)	233944 (99.96)
Orthopedic exam		
1 (yes)	10 (1)	14742 (6)
0 (no)	1774 (99)	219306 (94)
Disability		
0 (none)	1466 (82)	211280 (90)
1 (stretcher)	261 (15)	12491 (5)
2 (wheelchair)	57 (3)	10277 (5)
First triage visit		
1 (yes)	874 (49)	113617 (49)
0 (no)	910 (51)	120431 (51)
Glycemia (mg/dL)	163 (23-900)	147 (1-900)
Glycemia missing		
1 (yes)	1114 (62)	168300 (72)
0 (no)	670 (38)	65748 (28)
Abnormal glycemia		
1 (yes)	210 (12)	9314 (4)
0 (no)	1574 (88)	224734 (96)

The table shows number of patients. The figures in parentheses are the column percentages within each categorical variable for the respective outcome of admission.
