# Supplementary material for: Predicting Intensive Care Unit admission among patients presenting to the emergency department using machine learning and natural language processing
Source: PLoS One. 2020 Mar 3;15(3):e0229331. doi: 10.1371/journal.pone.0229331 (PMC7053743; doi:10.1371/journal.pone.0229331)
Supplement: S5 Table — The table shows number of patients. The figures in parentheses are the column percentages within each categorical variable for the respective outcome of admission. (PDF) [file pone.0229331.s007.pdf]

Table S5. Additional variables used for modelling both hospitals emergency departments data.

Variable (units)	BIDMC ICU		HBA ICU	
	Admission	No admission	Admission	No admission
Pain Scale				
0	1603 (47)	38305 (33)	475 (26.6)	43701 (18.7)
1	32 (1)	1885 (2)	21 (1.2)	2514 (1.1)
2	61 (2)	4474 (4)	57 (3.2)	8311 (3.6)
3	74 (2)	5141 (4)	75 (4.2)	13703 (5.9)
4	1002 (29)	15752 (13)	598 (33.5)	40367 (17.2)
5	120 (3)	8804 (8)	319 (17.9)	89875 (38.4)
6	102 (3)	7427 (6)	94 (5.3)	19606 (8.4)
7	106 (3)	9202 (8)	22 (1.2)	3775 (1.6)
8	135 (4)	11137 (10)	109 (6.1)	11204 (4.8)
9	58 (2)	5190 (4)	10 (0.6)	842 (0.4)
10	133 (4)	9906 (8)	4 (0.2)	150 (0.1)
Pain scale missing				
1 (yes)	1002 (29)	10222 (9)	542 (30)	25531 (11)
0 (no)	2424 (71)	107001 (91)	1242 (70)	208517 (89)
Number of missing vital signs and pain scale				
0	2612 (76)	109808 (93.7)	544 (30)	29263 (13)
1	279 (8)	4195 (3.6)	223 (13)	22238 (9)
2	81 (2)	796 (0.7)	112 (6)	14844 (6)
3	55 (2)	195 (0.2)	219 (12)	36322 (16)
4	31 (1)	118 (0.1)	162 (9)	20404 (9)
5	26 (1)	148 (0.1)	321 (18)	71656 (30)
6	342 (10)	1963 (1.7)	203 (12)	39321 (17)
Number of abnormal vital signs				
0	613 (18)	27149 (23.2)	545 (31)	137519 (58.8)
1	1017 (30)	47060 (40.1)	471 (26)	49918 (21.3)
2	962 (28)	29112 (24.8)	354 (20)	29620 (12.7)
3	582 (17)	12117 (10.3)	264 (15)	12629 (5.4)
4	209 (6)	1670 (1.4)	107 (6)	3809 (1.6)
5	41 (1)	111 (0.2)	43 (2)	553 (0.2)
Triage hour				
1am	111 (3)	2803 (2)	55 (3)	5352 (2)
2am	97 (3)	2319 (2)	45 (3)	4120 (2)
3am	66 (2)	1867 (2)	45 (3)	3429 (1)
4am	71 (2)	1542 (1)	29 (2)	3163 (1)
5am	67 (2)	1385 (1)	34 (2)	2941 (1)
6am	73 (2)	1590 (1)	35 (2)	3293 (1)
7am	71 (2)	2064 (2)	43 (2)	4252 (2)
8am	94 (3)	2992 (3)	79 (4)	7020 (3)
9am	120 (4)	4692 (4)	101 (6)	11485 (5)
10am	141 (4)	6231 (5)	102 (6)	15342 (7)
11am	210 (6)	7857 (7)	125 (7)	16572 (7)
12pm	184 (5)	7536 (7)	103 (6)	14215 (6)
1pm	222 (7)	7463 (6)	100 (6)	13072 (6)
2pm	188 (5)	7390 (6)	103 (6)	14640 (6)
3pm	188 (5)	7582 (6)	89 (5)	15412 (7)
4pm	190 (5)	7627 (7)	84 (5)	14216 (6)
5pm	191 (6)	7254 (6)	80 (4)	12618 (6)
6pm	195 (6)	7220 (6)	88 (5)	12257 (5)
7pm	214 (6)	6700 (6)	93 (5)	12564 (6)
8pm	163 (5)	6116 (5)	80 (3)	11431 (5)
9pm	156 (5)	5297 (5)	85 (5)	11749 (5)
10pm	148 (4)	4610 (4)	77 (4)	10461 (4)
11pm	156 (5)	3799 (3)	61 (3)	8153 (3)
12am	110 (3)	3287 (3)	48 (3)	6291 (3)
Triage weekday				
Monday	464 (13)	15049 (13)	260 (15)	37942 (16)
Tuesday	552 (16)	18120 (15)	264 (15)	35131 (15)
Wednesday	499 (15)	17369 (15)	246 (14)	34326 (15)
Thursday	481 (14)	16792 (14)	241 (13)	33994 (15)
Friday	506 (15)	16704 (14)	283 (16)	33580 (14)
Saturday	486 (14)	17514 (15)	237 (13)	29757 (13)
Sunday	438 (13)	15675 (14)	253 (14)	29318 (12)
Triage month				
January	296 (9)	10106 (9)	134 (8)	19278 (8)
February	253 (7)	9412 (8)	121 (7)	17586 (8)
March	326 (10)	10612 (9)	132 (7)	21702 (9)
April	229 (7)	8753 (7)	139 (8)	20204 (9)
May	261 (8)	8891 (8)	164 (9)	22297 (10)
June	331 (10)	10472 (9)	131 (7)	21811 (9)
July	285 (8)	10447 (9)	188 (11)	22218 (9)
August	228 (7)	8512 (7)	163 (9)	16929 (7)
September	218 (6)	8282 (7)	150 (8)	17497 (7)
October	314 (9)	10538 (9)	159 (9)	18301 (8)
November	373 (10)	11478 (10)	155 (9)	17762 (8)
December	312 (9)	9720 (8)	148 (8)	18463 (8)
Triage chief complaint (text)				

The table shows number of patients. The figures in parentheses are the column percentages within each categorical variable for the respective outcome of admission.
