# Supplementary material for: Predicting Intensive Care Unit admission among patients presenting to the emergency department using machine learning and natural language processing
Source: PLoS One. 2020 Mar 3;15(3):e0229331. doi: 10.1371/journal.pone.0229331 (PMC7053743; doi:10.1371/journal.pone.0229331)
Supplement: S7 Table — In brackets is the result for 100 bootstrapping iterations in 95% confidence intervals. (PDF) [file pone.0229331.s009.pdf]

Table S7. Average modeling performance results in test. In brackets is the result for 100 bootstrapping iterations in 95% confidence intervals.

Model	AUROC	AUPRC	Accuracy	Specificity	Sensitivity	PPV	F1-score	κ	Predictors
**BIDMC ICU**									
LR	0.78 [0.77-0.8]	0.09 [0.08-0.1]	0.61 [0.61-0.62]	0.60 [0.6-0.61]	0.88 [0.86-0.9]	0.06 [0.06-0.07]	0.11 [0.11-0.12]	0.06 [0.06-0.07]	ESI Priority
LR	0.86 [0.85-0.87]	0.20 [0.17-0.22]	0.78 [0.78-0.79]	0.78 [0.78-0.79]	0.79 [0.77-0.82]	0.1 [0.09-0.1]	0.17 [0.16-0.19]	0.13 [0.12-0.14]	ESI Priority + clinical variables
RFR	0.84 [0.83-0.86]	0.19 [0.17-0.22]	0.97 [0.97-0.97]	1.0 [1.0-1.0]	0.05 [0.03-0.06]	0.41 [0.32-0.48]	0.08 [0.06-0.1]	0.08 [0.06-0.1]	
RUSBoost	0.85 [0.84-0.86]	0.18 [0.16-0.2]	0.96 [0.95-0.96]	0.98 [0.98-0.98]	0.25 [0.23-0.28]	0.24 [0.22-0.26]	0.25 [0.22-0.27]	0.22 [0.2-0.25]	
LR	0.92 [0.91-0.93]	0.33 [0.29-0.35]	0.87 [0.87-0.87]	0.87 [0.87-0.87]	0.83 [0.8-0.85]	0.16 [0.15-0.17]	0.26 [0.25-0.28]	0.23 [0.21-0.24]	ESI Priority + clinical variables + Chief complaint (All)
RFR	0.9 [0.89-0.91]	0.28 [0.25-0.31]	0.97 [0.97-0.97]	1.0 [1.0-1.0]	0.06 [0.05-0.08]	0.52 [0.44-0.6]	0.11 [0.09-0.14]	0.1 [0.08-0.14]	
RUSBoost	0.89 [0.88-0.9]	0.24 [0.21-0.26]	0.96 [0.96-0.96]	0.98 [0.98-0.98]	0.25 [0.23-0.29]	0.31 [0.29-0.34]	0.28 [0.26-0.31]	0.26 [0.24-0.29]	
LR	0.91 [0.90-0.92]	0.32 [0.28-0.35]	0.86 [0.86-0.87]	0.86 [0.86-0.87]	0.81 [0.79-0.84]	0.15 [0.14-0.16]	0.25 [0.24-0.27]	0.22 [0.2-0.23]	All except priority
LR calibrated	0.91 [0.9-0.92]	0.30 [0.27-0.33]	0.86 [0.86-0.86]	0.86 [0.86-0.86]	0.82 [0.8-0.84]	0.15 [0.14-0.16]	0.25 [0.23-0.26]	0.21 [0.2-0.22]	
**HBA ICU&INT**									
LR	0.74 [0.72-0.76]	0.03 [0.02-0.03]	0.82 [0.82-0.82]	0.82 [0.82-0.83]	0.65 [0.61-0.68]	0.03 [0.02-0.03]	0.05 [0.05-0.06]	0.04 [0.03-0.04]	MTS Priority
LR	0.84 [0.82-0.85]	0.06 [0.05-0.07]	0.75 [0.75-0.76]	0.75 [0.75-0.76]	0.77 [0.73-0.8]	0.02 [0.02-0.03]	0.04 [0.04-0.05]	0.03 [0.03-0.03]	MTS Priority + clinical variables
RFR	0.81 [0.79-0.82]	0.05 [0.04-0.07]	0.73 [0.73-0.73]	0.73 [0.73-0.73]	0.73 [0.7-0.76]	0.02 [0.02-0.02]	0.04 [0.04-0.04]	0.03 [0.02-0.03]	
RUSBoost	0.82 [0.8-0.83]	0.06 [0.05-0.07]	0.98 [0.98-0.98]	0.98 [0.98-0.98]	0.22 [0.19-0.25]	0.09 [0.07-0.1]	0.12 [0.11-0.14]	0.11 [0.1-0.13]	
LR	0.86 [0.85-0.87]	0.08 [0.07-0.1]	0.83 [0.82-0.83]	0.83 [0.83-0.83]	0.72 [0.68-0.75]	0.03 [0.03-0.03]	0.06 [0.05-0.06]	0.05 [0.04-0.05]	MTS Priority + clinical variables + Chief Complaint (All)
RFR	0.81 [0.79-0.82]	0.05 [0.04-0.06]	0.74 [0.74-0.74]	0.74 [0.74-0.74]	0.73 [0.69-0.76]	0.02 [0.02-0.02]	0.04 [0.04-0.04]	0.03 [0.02-0.03]	
RUSBoost	0.81 [0.8-0.82]	0.05 [0.04-0.06]	0.97 [0.97-0.97]	0.98 [0.98-0.98]	0.24 [0.21-0.28]	0.08 [0.06-0.09]	0.11 [0.1-0.13]	0.1 [0.09-0.12]	
Multi-model	0.86 [0.84-0.87]	0.08 [0.06-0.09]	0.81 [0.8-0.81]	0.81 [0.8-0.81]	0.75 [0.72-0.78]	0.03 [0.03-0.03]	0.05 [0.05-0.06]	0.04 [0.04-0.05]	All
Multi-model	0.85 [0.83-0.86]	0.06 [0.05-0.07]	0.80 [0.8-0.81]	0.81 [0.8-0.81]	0.73 [0.7-0.76]	0.03 [0.03-0.03]	0.05 [0.05-0.06]	0.04 [0.04-0.04]	All except priority
Multi-model calibrated	0.85 [0.83-0.86]	0.06 [0.05-0.07]	0.84 [0.84-0.85]	0.85 [0.84-0.85]	0.68 [0.65-0.72]	0.03 [0.03-0.04]	0.06 [0.06-0.07]	0.05 [0.04-0.05]	

Abbreviations: ICU - Intensive Care Unit, ICU&INT - Intensive and Intermediate Care Units, AUROC - area under the ROC curve, PPV - positive predictive value, AUPRC - Area under the precision recall curve.
