# Supplementary figures and images for: Predicting Intensive Care Unit admission among patients presenting to the emergency department using machine learning and natural language processing
Source: PLoS One. 2020 Mar 3;15(3):e0229331. doi: 10.1371/journal.pone.0229331 (PMC7053743; doi:10.1371/journal.pone.0229331)

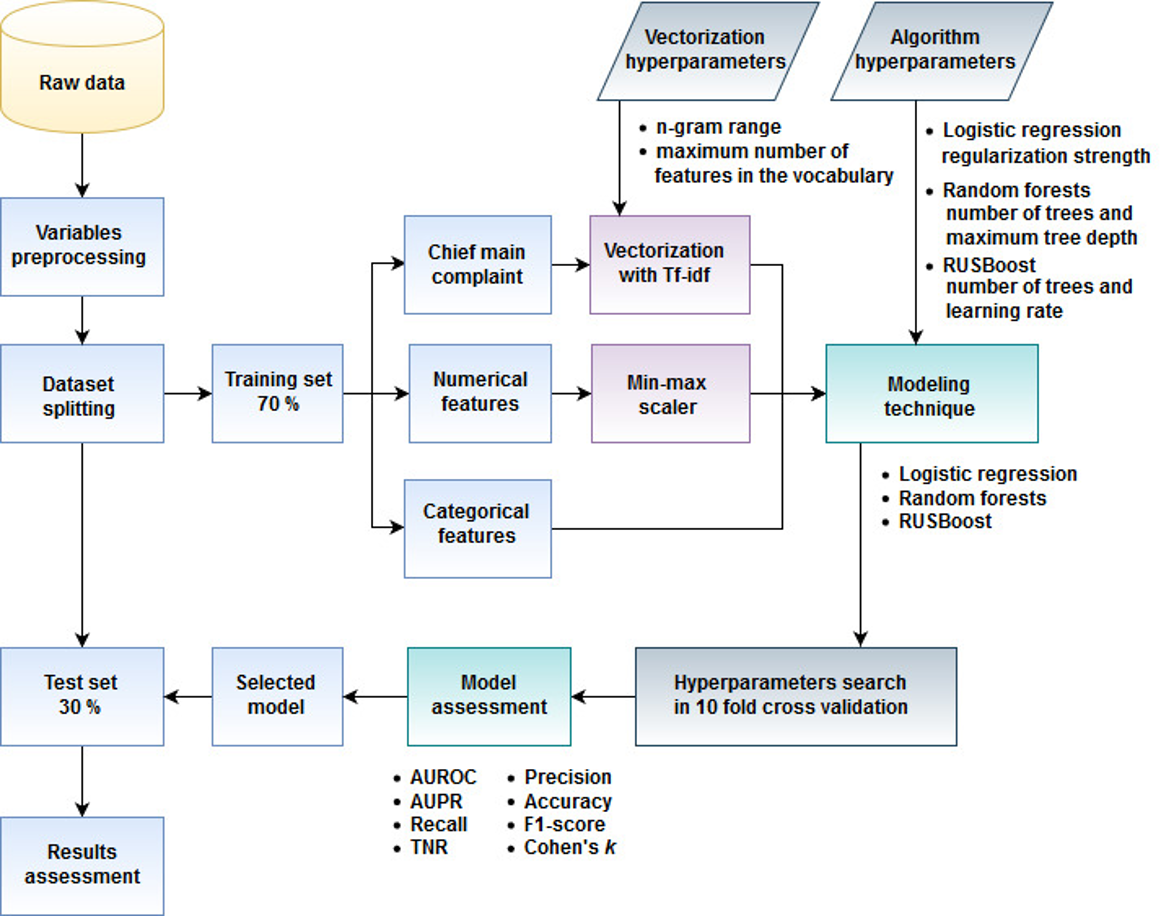

Supplement: S1 Fig — AUROC—area under the ROC curve, AUPRC—area under the precision recall curve, TNR—true negative rate or specificity. RUSBoost—Random undersampling boosting algorithm. Tf-idf—Term frequency–inverse document frequency. (TIF) [file pone.0229331.s010.tif]

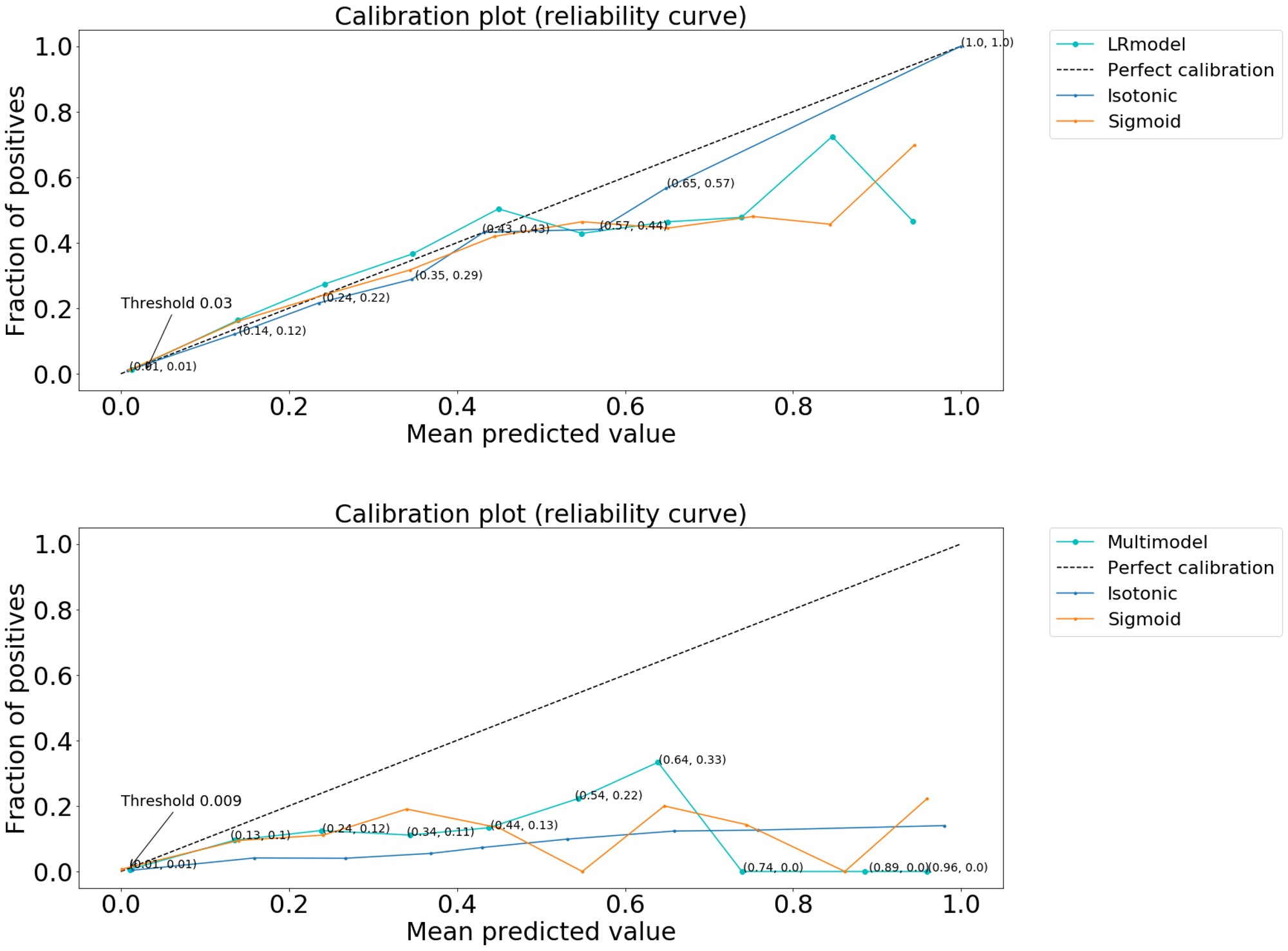

Supplement: S2 Fig — Annotations with labels are presented only for the selected models. (TIF) [file pone.0229331.s011.tif]

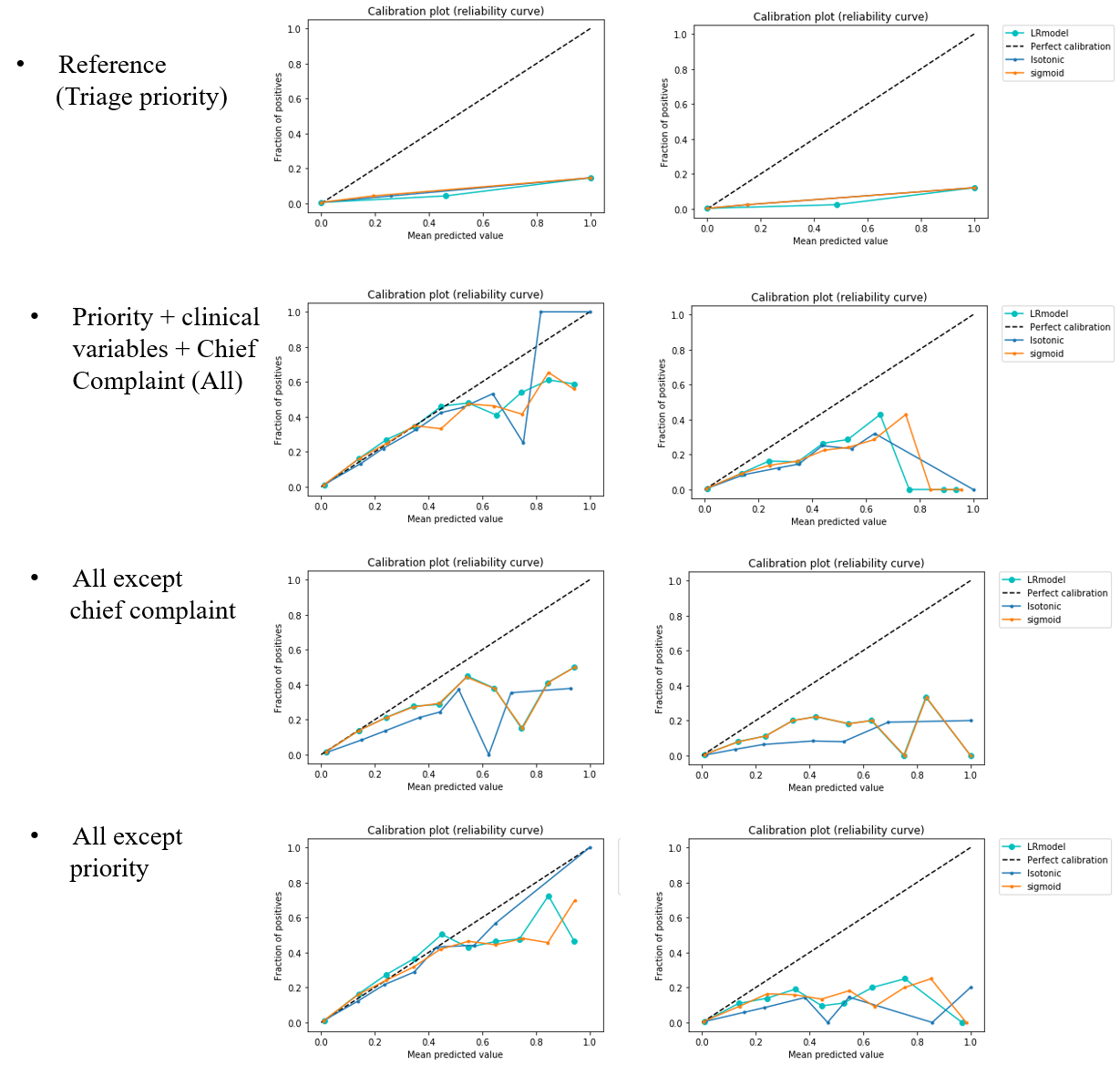

Supplement: S3 Fig — (TIF) [file pone.0229331.s012.tif]

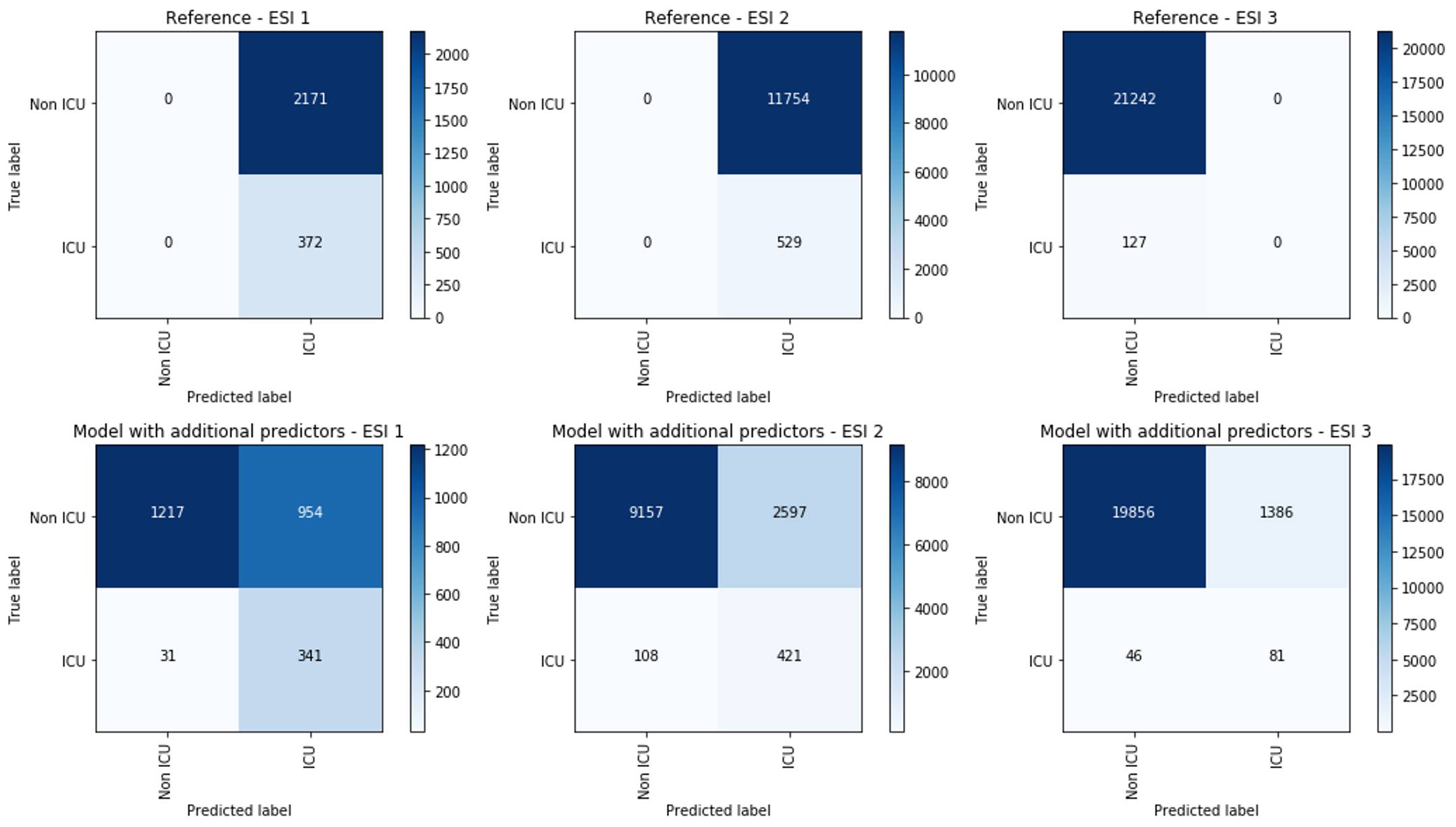

Supplement: S4 Fig — (TIF) [file pone.0229331.s013.tif]

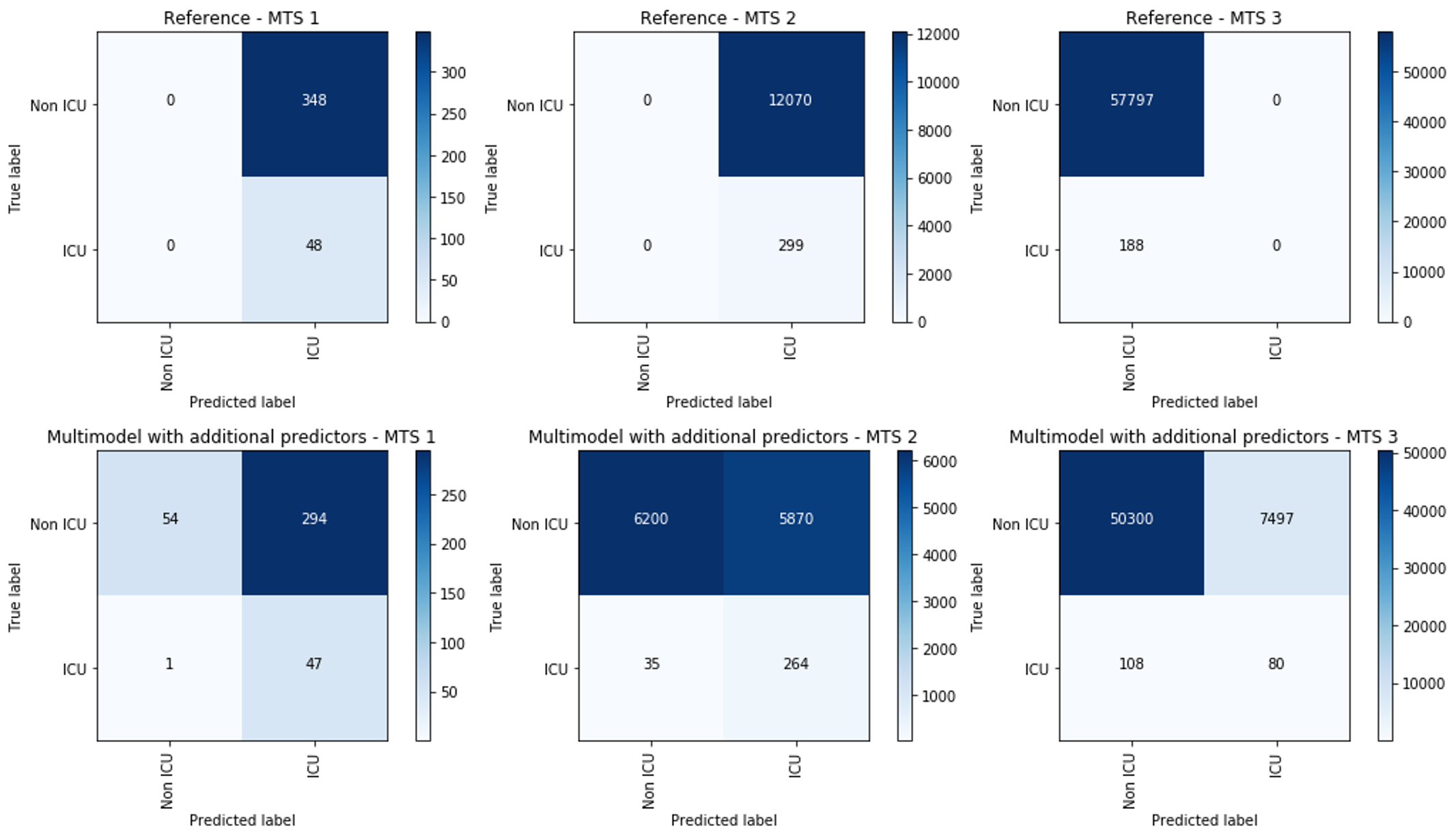

Supplement: S5 Fig — (TIF) [file pone.0229331.s014.tif]

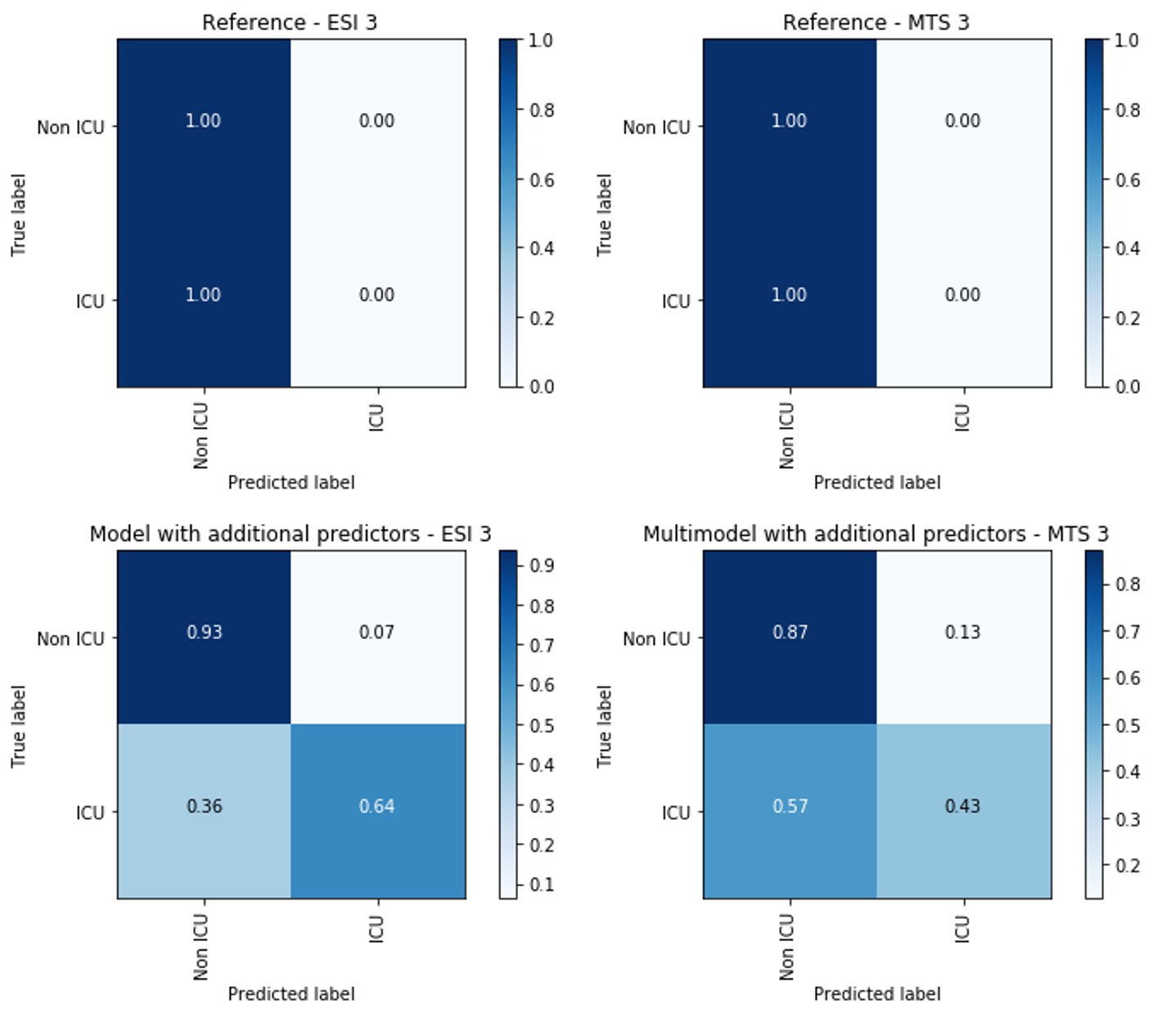

Supplement: S6 Fig — (TIF) [file pone.0229331.s015.tif]
